# Beyond risk reduction: Exploring the relation of cognitive control with adolescent positive and negative risk‐taking

**DOI:** 10.1111/jora.70103

**Published:** 2025-11-24

**Authors:** Hyeji Lee, Stella Haffner, Bonnie Auyeung, Nicolas Chevalier

**Affiliations:** ^1^ Department of Psychology Edinburgh UK; ^2^ Present address: Nuffield Department of Primary Care Health Sciences The University of Oxford Oxford UK

**Keywords:** adolescent, cognitive control, effortful control, executive function, risk‐taking

## Abstract

Taking risks is a crucial part of adolescent development, encompassing both positive (socially valued) and negative (potentially life‐threatening) behaviors. While cognitive control is known to reduce harmful risk behaviors, its relationship with beneficial risk‐taking remains unclear. This study investigated how multiple components of cognitive control relate to both types of risk‐taking and explored learning as a potential pathway to adaptive risk‐taking. We assessed 127 adolescents (ages 12–18, 65% female, 60% White) using experimental cognitive tasks, self‐report measures, and an adapted balloon analog risk task. Working memory and proactive control were associated with reduced negative risk‐taking (NRT) but not positive risk‐taking (PRT). Effortful control showed a unique divergent pattern, being associated with both reduced NRT and increased willingness for PRT. These associations diminished with age, perhaps due to the increasing influence of external factors like opportunity and social context. Better learning in low‐risk experimental conditions related to reduced real‐world NRT, though this learning ability was not associated with cognitive control measures. These findings contribute to expanding our understanding of how cognitive control relates to adolescent adaptive risk‐taking and open up perspectives for effective interventions.

## INTRODUCTION

Risk‐taking propensity in adolescence has received considerable attention because it can lead to life‐threatening consequences (Boyce et al., 2008; Savolainen et al., 2012; Steinberg, 2004). It has been characterized as maladaptive behavior from early pioneering studies to more recent work (Gardner & Steinberg, 2005; Shulman et al., 2016). Recently, however, the benefits of risk‐taking have started to get recognition with risk‐taking being viewed as a pivotal part of a successful transition to adulthood (Ciranka & van den Bos, 2021; Crone & Dahl, 2012; Do et al., 2020; Duell & Steinberg, 2019; Romer et al., 2017; Telzer et al., 2018).

Not all risk‐taking is inherently harmful or life‐threatening, and indeed some risky behaviors can be viewed as adaptive (Fischer & Smith, 2004; Romer et al., 2017), prosocial (Do et al., 2017), and positive (Duell & Steinberg, 2019, 2021). Positive risk‐taking (PRT) refers to actions that potentially benefit adolescent well‐being and development and are both legal and socially acceptable (e.g., taking a challenging class or asking someone out on a date). Conversely, negative risk‐taking (NRT) refers to actions that are mostly detrimental to adolescent well‐being and development and are antisocial or sometimes illegal (e.g., speed driving or unsafe sex). While both PRT and NRT entail uncertainty and the potential for an undesirable outcome, they differ in their developmental benefits and social acceptability (Duell & Steinberg, 2021).

Previous research has found a moderate positive correlation between PRT and NRT (*r* = .20 to .31; Duell & Steinberg, 2020; Patterson et al., 2022), suggesting that individuals who engage in one type of risk‐taking also tend to engage in the other, which is likely driven by a shared underlying risk propensity. Studies have also identified both shared and unique psychological correlates such as sensation seeking, future time perspective, tolerance to ambiguity, and sensitivity to reward that are common predictors of both risk types, while impulsivity uniquely associates with NRT and sensitivity to punishment shows differential patterns between the two (Fryt et al., 2023; Duell & Steinberg, 2020; Patterson et al., 2022). Although these findings have revealed important aspects of the psychological profiles underlying different risk‐taking patterns, they have not addressed how different aspects of cognitive control may be involved in both PRT and NRT, and whether cognitive control may influence risk‐taking by supporting learning in risky contexts. Addressing these questions could inform interventions designed to promote positive risks while discouraging harmful ones. To address this gap, the current study employed comprehensive experimental tasks alongside questionnaires to expand our understanding of cognitive control processes that influence adolescent adaptive risk‐taking.

### Understanding the role of cognitive control in adaptive risk‐taking during adolescence

Traditionally, dual‐process models have been used to account for adolescent risk‐taking. These models posit an imbalance between a heightened reward sensitivity system (involving subcortical regions such as the ventral striatum) and a relatively immature cognitive control system (supported by the frontoparietal network) (Casey et al., 2008; Somerville & Casey, 2010; Steinberg, 2008). Risk‐taking is thought to arise from reward sensitivity overwhelming the cognitive control system during adolescence (Shulman et al., 2016), and the models have been supported by extensive neurobiological evidence (i.e., Galvan et al., 2006; Qu et al., 2015).

The dual‐process models assume that cognitive control always operates in opposition to the reward system. In situations involving NRT, where an immediate reward might lead to long‐term harm, the model suggests that strong cognitive control should suppress the urge to pursue that reward. This explanation fits well for scenarios requiring restraint from impulsive behaviors. However, it does not fully explain PRT that involves taking actions despite potential risks (e.g., taking a challenging class despite the possibility of failure), as reward sensitivity can be a driving force of PRT (Duell & Steinberg, 2020; Romer et al., 2016). Similarly, cognitive control may support PRT by allowing adolescents to make thoughtful decisions, commit to risky goals, and take the necessary actions to reach them. Consistently, risk‐taking in ambiguous situations or involving strategic exploration and effort may require an *increase* in cognitive control (Do et al., 2020; Romer et al., 2017). Another key difference between PRT and NRT lies in the timing of rewards. While NRT tends to provide immediate rewards, PRT involves an initial period of effort before later benefits emerge. For example, choosing a challenging class may feel daunting at first, yet it ultimately pays off in greater achievement and intrinsic satisfaction. Consequently, the interplay between cognitive control and reward processing in PRT may diverge markedly from that in NRT, suggesting that current dual‐system models may not fully capture these complex interactions.

Nonetheless, unlike the well‐established negative relation between cognitive control and NRT, only a few studies have explored the relation between cognitive control and PRT, and they have reported null findings (Duell & Steinberg, 2020; Fischer & Smith, 2004). These studies have focused on inhibitory control, that is, the ability to suppress impulses or distractions that might interfere with goal‐directed behavior. While the role of inhibitory control in risk‐taking aligns well with dual‐process models explaining NRT, where adolescents must refrain from harmful impulses, it falls short when accounting for PRT. Therefore, to understand the link between cognitive control and different types of risk‐taking during adolescence, it is crucial to examine its various components.

Cognitive control is not solely about suppressing impulses but also encompasses the capacity to make thoughtful decisions and commit to effortful actions to achieve one's goals. Importantly, complex behaviors such as planning and strategy selection, which are involved in PRT, rely on multiple components of cognitive control (i.e., working memory, inhibitory control, cognitive flexibility) working in concert (Diamond, 2013). Engaging in positive risks requires adolescents to actively take actions that are often both effortful and uncertain such as strategic exploration or pursuing ambitious goals. These actions likely require maintaining goal‐relevant information in working memory while flexibly adjusting strategies based on environmental feedback and changing circumstances, functions that point directly to the importance of working memory and cognitive flexibility, rather than just inhibitory control. Importantly, the respective influence of these cognitive control components on risk‐taking may change with age during adolescence, as seminal work has shown that mature cognitive control engages each component differently depending on specific task demands (Miyake et al., 2000), and this multi‐component structure emerges during adolescence (Lee et al., 2013).

Another critical development in adolescent cognition is the qualitative shift in how cognitive control is deployed. As cognitive control matures, a key developmental change is the shift from a reactive to a more proactive mode of engagement (Chevalier & Blaye, 2022). Reactive control involves simply reacting to conflicts as they arise, whereas proactive control involves anticipating and preparing for upcoming demands in advance (Braver, 2012). Proactive control develops throughout adolescence (e.g., Andrews‐Hanna et al., 2011) and is related to planning and future‐oriented thinking (Andrews‐Hanna et al., 2011; Mahy & Munakata, 2015). The future‐oriented nature of proactive control makes it a potential candidate for explaining adaptive risk‐taking. Proactive control may help adolescents plan actions and anticipate their outcomes, thus both facilitating PRT and avoiding NRT by enabling individuals to think about the future consequences of their actions and adjust their behavior accordingly. Although the relationship between proactive control and risk‐taking during adolescence has not been directly addressed, some findings are consistent with this potential role. Adolescents with delinquent histories struggled more with implementing proactive control (Iselin & Decoster, 2009), while youth at risk for anxiety showed fewer anxiety issues when they had higher levels of proactive control, suggesting a protective role in emotional regulation (Valadez et al., 2022).

### Understanding the role of effortful control in adolescent adaptive risk‐taking

To better understand the role of cognitive control in adolescent risk‐taking, it is necessary to consider how it is deployed in emotionally charged, social contexts, as the response of the reward system is strongest in such contexts during adolescence (Pfeifer & Allen, 2021). The construct of effortful control, which overlaps significantly with cognitive control in terms of its conceptual framework and its neural correlates (Zhou et al., 2012), emphasizes the temperamental component of cognitive control as well as how individuals manage emotions and behavior in emotionally and motivationally charged scenarios. As such, it captures cognitive control as it operates in the real life of adolescents. Lower effortful control in early adolescence predicts NRT behaviors, such as the initiation and escalation of substance use (Peeters et al., 2017), and delinquent and aggressive behavior (King et al., 2013; Wang et al., 2015). While no research has investigated the association between effortful control and PRT, there are reasons to suspect that greater effortful control may foster PRT.

Conceptually, effortful control may help adolescents to focus on difficult tasks and manage emotional responses in stressful situations. Effortful control enables adolescents to focus on challenging tasks while simultaneously managing the emotional responses that arise in stressful or novel situations. This capacity is particularly relevant to PRT, which often requires individuals to override natural tendencies toward avoidance or anxiety when confronted with unfamiliar but potentially beneficial opportunities. In line with this, adolescents with higher effortful control are better equipped to buffer against negative emotional states (Youssef et al., 2016) or environmental stressors (Oldehinkel et al., 2011) and are known to have better emotional regulation (Kanske & Kotz, 2012) and social competence (King et al., 2013).

### Learning as a pathway to adolescent adaptive risk‐taking

During adolescence, individuals frequently encounter novel situations where outcomes are uncertain due to limited prior experience (Hartley & Somerville, 2015). As adolescents explore and learn from their environment, they selectively engage in more calculated risks in order to avoid severe consequences (Ciranka & van den Bos, 2021; Romer et al., 2017). In other words, in contexts that allow exploration and learning, adolescents can engage in reasoned risk behavior, which involves strategic, planned, and deliberate decisions to take risks for potential benefits (Ciranka & van den Bos, 2021; Humphreys et al., 2013; Romer et al., 2017). This adaptive learning process helps them reduce uncertainty and adjust their behavior to new environments (Crone & Dahl, 2012).

Several studies have shown that adolescents can indeed engage in beneficial risk‐taking in settings where they can explore and learn from the environment (e.g., Blair et al., 2018; Ciranka & van den Bos, 2021), however, it remains unclear whether an adolescent's capacity for learning within a risky task transfers to real‐world contexts of adolescents. Much of the existing research has focused on the link between general risk task performance and real‐world risk‐taking, especially regarding NRT (Defoe et al., 2015), with less attention paid to how the process of learning itself relates to adaptive outcomes, such as engaging in positive risks and avoiding negative ones.

Importantly, the learning process in risk‐taking tasks may provide a potential mechanism for the association between cognitive control and adaptive risk‐taking behavior. Cognitive control is critical for learning from feedback and updating strategies in response to changing outcomes, resulting in better performance in risk‐taking scenarios (Blair et al., 2018; Ogilvie et al., 2020). This suggests a plausible but underexplored pathway; rather than simply suppressing risk, cognitive control may promote adaptive risk‐taking by facilitating the learning process that allows adolescents to distinguish between beneficial and detrimental risks.

## THE PRESENT STUDY

The present study investigated the underlying cognitive processes that drive adaptive risk‐taking during adolescence through four main research questions. First, to understand the role of cognitive control in adolescent adaptive risk‐taking, we employed a comprehensive assessment of cognitive control components including working memory, cognitive flexibility, inhibitory control, and proactive control. Building on prior relevant findings and conceptual relations discussed in the introduction, we generally expected that better cognitive control capacity would be related to adaptive risk‐taking in real life (i.e., less NRT and more PRT). Second, we examined whether and how effortful control, as a measure of cognitive control applied in emotionally charged, real‐life contexts, may relate to NRT and PRT. Based on the conceptual framework that effortful control helps adolescents focus on difficult tasks and manage emotional responses in stressful situations, we hypothesized that higher effortful control would be associated with less NRT and more PRT. Third, using an adapted version of the balloon analogue risk task (BART), we examined whether adaptive risk‐taking in experimental contexts relates to real‐world risk‐taking behaviors. BART, which was originally designed to assess risk‐taking in clinical populations with substance use disorders (Lejuez et al., 2002), has been extensively used in adolescent risk‐taking research (e.g. Braams et al., 2015; Lejuez et al., 2003). The task involves participants inflating a virtual balloon, earning money with each pump but risking the loss of all accumulated earnings if the balloon bursts. While this task has yielded mixed findings regarding its association with NRT (Lauriola et al., 2014) and shown no relation between performance on risk‐taking tasks with PRT (Duell & Steinberg, 2020), the BART remains an effective tool for simulating real‐life adaptive risk‐taking scenarios, where taking moderate risks is beneficial but excessive risks lead to negative outcomes (Lejuez et al., 2002). Also, unlike previous studies that primarily focused on the number of pumps which primarily reflects individual risk‐taking tendencies, we focused on changes in this behavior across trials that highlight learning and adaptive decision‐making. Additionally, we adapted the BART to be more conducive to adolescent learning, providing opportunities to maximize rewards through successful learning under risk. We hypothesized that better learning capability on the BART would be associated with less NRT and more PRT. Lastly, given that cognitive control may enable more effective learning in uncertain environments and facilitate adaptive strategy adjustment, we investigated the association between cognitive control and BART performance. We hypothesized that higher cognitive control abilities would be associated with better learning performance on the BART.

Further, given our wide age range spanning early to late adolescence (12–18) and that age is highly related to opportunity and access to risk‐taking during adolescence (Defoe et al., 2015; Shulman et al., 2016), age was included as a key variable in all analyses on an exploratory basis, with no specific hypothesis regarding age effects. Critically, PRT and NRT were measured via self‐reports, which have shown strong reliability (Duell & Steinberg, 2020; Fryt & Szczygiel, 2021). In addition to asking if participants had previously engaged in PRT behaviors, we also inquired whether they would be willing to engage in them if provided the chance. This approach allowed us to assess participants' willingness for PRT regardless of their past or present accessibility to such opportunities. This was especially important given that the opportunities for PRT are highly dependent on the adolescents' specific environment, as these behaviors are socially desirable and often typically occur within educational settings. The educational context itself can vary significantly based on the socioeconomic background of the participants. For example, access to advanced classes and extracurricular activities can be limited for low‐income and minority students (Kolluri, 2018) or students in rural schools (Snellman et al., 2015).

## METHODS

### Participants

A total of 127 participants completed the online study. Participants were recruited in the United Kingdom through social media, online parenting groups, and word of mouth, as well as from first‐year students at the University of Edinburgh (aged 12–18 years, *N* = 127). Six participants were excluded due to failing attention check questions in the questionnaire. Additionally, 13 participants were excluded from analysis based on reaction time and accuracy across cognitive control measures and the BART. Specific exclusion criteria are detailed in the subsection below (see Self‐report measures and Experimetnal measures). As a result, 108 participants aged 12–18 years old were included in the final analysis (*M* = 15.72 years, SD = 1.74, female = 65). The distribution by age was 12 (*n* = 7), 13 (*n* = 11), 14 (*n* = 7), 15 (*n* = 11), 16 (*n* = 30), 17 (*n* = 28), and 18 years (*n* = 14). Most participants reported their parental education as being at the college or university level or higher (70.37%). In terms of ethnicity, most participants were White (60.19%), followed by Asian/Asian British (22.22%) and Black/Black British (10.19%), with the remaining participants identifying as Mixed or from other ethnic backgrounds.

### Procedure

Participants registered via an online application form and received instructions via email, along with scheduling for a video call. For participants under 16, parental presence was required, and parents completed a short demographic questionnaire and provided consent for their child's participation. After receiving instructions via a video call, participants were given a link to the online experimental platform (Gorilla). The tasks were designed in a gamified format with audio‐guided instructions to enhance engagement. While participants performed the tasks, experimenters were available for assistance but did not directly observe participants while they completed tasks independently. To ensure participants' engagement and to prevent random responses, we informed them that insufficient attention would impede their progress to the next screen and extend the task duration. Also, during the initial stage of the questionnaire, if participants responded too quickly (suggesting inattention), a warning screen appeared with an alarm sound. The experiment consisted of four stages: (1) a demographic questionnaire, (2) a cognitive control task battery, (3) risk‐taking and effortful control questionnaires, and (4) the adjusted BART. Upon completion of all stages, participants were compensated with a £10 Amazon gift card. An experiment overview is in Figure S4. Participants recruited at the university received course credits. This study was approved by the University of Edinburgh ethics board (reference number: 421‐2122/6).

### Self‐report measures

#### Risk‐taking

PRT and NRT was assessed using an adaptation of a scale developed and psychometrically validated by Duell and Steinberg (2020). The original scale demonstrated strong psychometric properties, including good construct validity (CFI = 0.97, RMSEA = 0.05), measurement invariance across age and gender, and convergent/discriminant validity with sensation seeking, impulse control, and school engagement (Duell & Steinberg, 2020). The 14 negative items captured contemporary adolescent behaviors such as riding in a car with an intoxicated driver and cheating on a homework assignment or exam. We made a minor adaptation to include vaping behavior alongside smoking. Participants were asked how many times they had engaged in each behavior over the past 6 months (0 = Never, 1 = Once, 2 = Sometimes, 3 = Often, 4 = Very Often). We also calculated a variety score for NRT (*NRT_done*) by coding responses as dichotomous variables (0 = Never, 1 = at least once). Participants could also choose not to report their behavior by selecting “prefer not to say” (Cronbach's α = 0.71).

The 14 positive items reflected daily activities that adolescents are likely to engage in, encompassing social (e.g., starting a new friendship), academic (e.g., taking a challenging class), and extracurricular (e.g., trying a new sport) aspects of life. Each behavior was described with the uncertainty of possible outcomes to highlight its riskiness (e.g., “stood up for what you believe is right, even though you thought someone might disagree with you”). In contrast to the original scale, which asked about the frequency of these behaviors over the past 6 months (0 = never to 4 = more than five times), our study only asked whether participants had ever engaged in the activity (coded 1 for “yes” and 0 for “no”), to calculate variety scores, the proportion of behaviors that have been done at least once to all behaviors, which is a common measure employed in risk‐taking research. This approach avoids potentially misleading frequency reports, which can vary greatly depending on adolescents' opportunities to engage in such behaviors (Duell & Steinberg, 2020).

In addition to Duell and Steinberg's scale, we asked whether participants would try the PRT behaviors if given the chance. We introduced these additional items because PRT is highly context‐dependent and often influenced by access to resources and support. For example, some adolescents have greater access to advanced classes and extracurricular activities, whereas others do not. As a result, we had two PRT measures: past PRT (*PRT_done*), and willingness for PRT (*PRT_will*). Participants responded to two questions per item (“Have you engaged in this activity during the last 6 months?” and “Would you try it if you had the chance?”), with options to answer “yes”, “no” or “prefer not to say”(Cronbach's *α* = 0.68 for PRT_will, 0.6 for PRT_done respectively). Of note, we elected not to collect willingness for NRT for several reasons. First, there is a fundamental ethical difference between asking about past records versus future willingness. Questions about past NRT address already established facts, while asking about willingness to engage in future NRT in an undecided future carries the potential for intervention. Additionally, while PRT is something that adolescents can plan and prepare for in the future (e.g., enrolling in challenging courses, trying out for an audition), most negative behaviors occur in the heat of the moment (e.g., riding in a car with an intoxicated driver, having unprotected sex) and are thus not likely to be planned ahead. Therefore, measuring willingness for NRT may not accurately capture the propensity for such behaviors, as these actions are rarely the result of prospective decisions.

#### Effortful Control

Effortful control was measured using the “Effort control” subscale of the Early Adolescent Temperament Questionnaire revised (EATQ‐r; Putnam et al., 2001). This factor assesses self‐regulation and executive functioning in adolescents' daily lives (Putnam et al., 2001). It consists of three components: attention (the ability to focus and shift one's attention at will, e.g., “It is easy for me to really concentrate on homework problems.”), activation control (the ability to perform an action despite a strong tendency to avoid it, e.g., “If I have a hard assignment to do, I get started right away.”), and inhibitory control (the ability to plan and suppress inappropriate responses, e.g., “The more I try to stop myself form doing something I shouldn't, the more likely I am to do it.”). Each item is answered on a 5‐point Likert scale (1 = *Almost always untrue of you*; 5 = *Almost always true of you*). The final score was calculated by averaging scores following the original questionnaire instructions (Cronbach's *α* = 0.85). An attention check question was included in the middle of the questionnaire, resulting in the exclusion of data from 6 participants.

#### Reward/punishment sensitivity

Reward and Punishment sensitivity was assessed using the Behavioral Inhibition System and the Behavioral Approach System questionnaires (BIS/BAS; Carver & White, 1994). The BIS/BAS questionnaire consists of four subscales: BAS Drive (a measure of persistence in the pursuit of goals), BAS Fun seeking (a measure of desire for rewards and the willingness to approach rewards), BAS Reward responsiveness (a measure of responses to rewards and reward anticipation), and BIS (a measure of punishment sensitivity). Participants responded using a 4‐point Likert scale to indicate the extent to which each statement applied to them (1 indicating *very false* and 4 indicating *very true*). We use the sum of the BAS components as reward sensitivity and the BIS subscale as punishment sensitivity, with higher scores indicating greater sensitivity. Internal consistency was acceptable to good for the BIS/BAS subscales: BIS (*α* = .82), BAS Drive (*α* = .82), BAS Reward responsiveness (*α* = .70), and BAS Fun seeking (*α* = .61).

### Experimental measures

#### Working memory

Working memory was measured with the Backward Digit Span Task adapted from Chere and Kirkham (2021). Participants were presented with a sequence of numbers and asked to recall them in reverse order. The task began with two‐digit sequences and increased by one digit for each subsequent level. Each level consisted of five trials, with participants required to correctly answer three out of five to progress. The task ended if a participant made three errors within a single level. Each trial began with a 450 ms fixation cross, followed by single number presentations lasting 1500 ms each, with 500 ms intervals. Numbers were displayed in a pseudo‐random order, ensuring that consecutive digits were not identical. To familiarize participants with the procedure, two practice trials with performance feedback were provided. There was no feedback during the actual trials. The task measured the total number of correct trials (final score) and the overall proportion of correct trials throughout the assessment and we used the final score for the analysis. Data from two participants were excluded because they did not follow the instructions and reported numbers in order instead of in reverse order.

#### Inhibitory Control

Inhibitory control was measured using the Flanker task, adapted from Anwyl‐Irvine et al. (2020) and Rueda et al. (2004). Participants had to press on the button of the same side as the central target while ignoring the flanking distractors. The task began with 12 practice trials, during which feedback was provided after each response. The main task consisted of three blocks, each containing 24 trials, for a total of 72 trials. In each trial, participants were presented with five arrows displayed centrally on the screen. The middle arrow served as the target, while the surrounding arrows acted as flankers. In congruent trials, all arrows pointed in the same direction, whereas in incongruent trials the flanking arrows pointed in the opposite direction of the target arrow. Between trials, a central fixation cross appeared for a variable duration (400, 600, 800, or 1000 ms). The direction of the central arrow (left or right) and the congruence of the surrounding arrows were counterbalanced across trials. Both trial order and fixation cross timing were randomized for each participant. Participants with an overall accuracy rate below 60% were excluded from further analysis, resulting in 7 participants being rejected. This criterion followed the methodology of Chere and Kirkham (2021) but adopted a more conservative threshold than their 50% criterion to ensure task engagement. Trials with reaction times above or below 2.5 standard deviations from each participant's mean (calculated across all conditions) were removed. Additionally, any trials with response times faster than 100 ms were excluded from the analysis (7.63% in total). For data analysis, reaction times were log‐transformed to address skewness in the distribution. The congruency effect was calculated by subtracting the mean reaction time for correct congruent trials from the mean reaction time for correct incongruent trials.

#### Cognitive flexibility and proactive control

Cognitive flexibility and proactive control were assessed using a cued task‐switching paradigm consisting of four blocks. See Figure S5: two single‐task blocks (Shape and Color) followed by two mixed‐task blocks (reactive and proactive). Participants switched between shape‐ (square vs. circle) and color‐matching (blue vs. red) tasks. Each single‐task block included 4 practice trials and 12 test trials. In each trial, a fixation cross was displayed for 350 ms, followed by the word “Ready” for 1500 ms before the stimulus appeared. The stimulus was presented in the center of the screen, with response instructions (“Press F for Square”, “Press J for Red”) displayed below it on the left and right. This screen remained visible for 1500 ms. After participants responded, feedback was given for 200 ms. In the mixed‐task blocks, both the Shape and Color tasks were presented, requiring participants to switch between task rules based on cues. Cognitive flexibility was measured using switching costs. Switching costs, representing the cognitive cost of switching between tasks, were calculated by subtracting mean reaction times of repeat trials (same task as the previous trial) from switch trials (different task from the previous trial) within each mixed block. For proactive control assessment, two types of mixed blocks were implemented. In the proactive mixed block, the cue (“Shape” or “Color”) was presented 1500 ms ahead of target onset, allowing participants to proactively prepare for the upcoming task. In the reactive mixed block, the word “Ready” (1500 ms) preceded the simultaneous presentation of the cue and stimulus, so that proactive preparation before target onset was not possible. Thus, proactive control engagement should lead to faster responses in the “proactive” than in the “reactive” blocks, while reactive control engagement would result in similar RTs across blocks. Following prior studies (e.g., Kubota et al., 2020), proactive control was quantified by subtracting the mean reaction times in the proactive blocks from the reaction times in the reactive blocks. Higher values indicated better proactive control. Each mixed block contained 48 trials, equally divided between Shape and Color tasks, presented randomly with a maximum of three consecutive same‐task trials. Participants with accuracy rates below 60% were excluded from the analysis (2 participants). For analysis, only correct responses were considered, with outliers (reaction times >*M* + 3*SD* or 10,000 ms, and <*M* −3*SD* or 200 ms, 3.17% of total trials) removed. Response times were log‐transformed to address skewness.

#### BART performance

We adapted the original version of the BART (Lejuez et al., 2002). Our adaptation involved two balloons with different explosion probabilities to allow participants to learn the explosion probability distribution. Prior studies have shown that participants' performance improved in later trials, suggesting a better grasp of the probability distribution of different balloons over time (Blair et al., 2018; Lejuez et al., 2002). Our focus was on how participants would learn about the environment and adapt their behavior accordingly. To do so, they needed to explore the number of pumps that would cause balloons to burst and learn the explosion probabilities as a function of pump numbers to optimize rewards while minimizing losses with the control.

Following the original BART design (Lejuez et al., 2002), we implemented two conditions: a low‐risk (1–128 range, average explosion point 64) and a high‐risk (1–32 range, average explosion point 16) condition. However, unlike previous studies, all balloons were the same color and the task was divided into two blocks of 20 balloons each. Participants completed the low‐risk condition first, followed by the high‐risk condition, without knowledge of the explosion probabilities. This approach allows clear observation of pumping behavior dynamics across trials in each condition, minimizing trial‐dependent stochasticity. We maintained a consistent order of trials and conditions across participants to reduce redundant variability as prior research showed that performance on the BART is strongly influenced by external stochasticity (e.g., the number of early bursts, trial sequences), which could impair accuracy in assessing participants' decision‐making process (Buelow & Blaine, 2015). The order was determined randomly by a program while keeping average explosion points of 64 and 16 within 20 trials for each condition, and we adhered to that sequence throughout the entire session. Additionally, the relatively stable condition allowed participants to learn and make reasoned risky decisions (Humphreys et al., 2013), which was of interest here. We expected a linear trend in both conditions if participants learned the explosion probability successfully. In the low‐risk condition, where the explosion probability was significantly low (average explosion point around 64), participants needed to pump extensively to discover the closest explosion point. This effort under uncertainty was crucial for accurately estimating the explosion probability. In contrast, in the high‐risk condition, where the balloon frequently exploded early (average explosion point around 16), participants had to quickly adapt their strategy to the higher explosion probability and reduce their pumping behavior to minimize unnecessary explosions while getting a reward.

We used standard BART measures, including the mean pump of unexploded balloons and the number of exploded balloons (Lejuez et al., 2002), with higher scores indicating greater risk‐taking propensity. While the number of pumps primarily reflects individual risk‐taking tendencies, changes in this behavior across trials highlight learning and adaptive decision‐making. To assess these behavioral changes, we adapted the “pump ratio” index from Blair et al. (2018), which compares pumps in the second half of the task to the first half for each risk condition, to examine general behavioral trends of all participants. However, this index can be heavily influenced by initial pumping behavior and individual risk propensity, making it difficult to compare learning processes across participants. To address this limitation and more accurately measure individual behavior change independently of initial risk tendencies, we applied a linear mixed model to analyze change in the number of pumps across trials and estimate individual learning slopes (i.e., how fast each participant learned the probability of balloon burst and adjust their behavior after each additional trial). Model results are in Table S4. These learning slopes showed significant correlations with total points: positive in low‐risk trials (*r* = 0.30, *p* < .01) and negative in high‐risk trials (*r* = −0.42, *p* < .001), indicating that participants who showed better learning earned more points overall. Results are presented in Figure S3. We excluded three participants because over 80% of their reaction times were under 50 ms.

### Data analysis

#### Associations between control variables and PRT and NRT

To examine if cognitive control and effortful control may be differently associated with PRT and NRT behavior, a series of linear mixed effect models were performed. Each model included a three‐way interaction between risk type, age, and one of the following: working memory, inhibitory control, cognitive flexibility, proactive control, and effortful control. As age relates to both cognitive control abilities and risk‐taking behavior during adolescence (Shulman et al., 2016; Tervo‐Clemmens et al., 2023), age was included as an interaction term to examine whether the relationship between cognitive control and risk‐taking behavior may vary with age. In each model, reward sensitivity and punishment sensitivity were included as covariates. Participant ID was incorporated as a random effect. While dual‐process models typically examine interactions between cognitive control and reward sensitivity, we controlled for these motivational factors rather than testing their interactions for two reasons: first, limited research on PRT presents inconsistent findings, making it difficult to formulate directional hypotheses; second, our model was designed to compare PRT and NRT simultaneously, and adding another interaction term would create excessive complexity beyond our primary focus in this study. Model results are presented in Table S1. For the outcome variable, the data were transformed into long format, and a new variable named ‘Behavior Propensity’ was created, which includes all kinds of self‐reported risk‐taking (a variety score), and another new categorical variable ‘Risk Type’ which can tell the type of risk behavior. NRT was the reference level for the model (past NRT: *NRT*, willingness for PRT: *PRT_will*, past PRT: *PRT_done*). This allowed us to examine if there was an interaction effect between components of cognitive control and risk type on behavior propensity. We ran the same model on each different component of cognitive control (e.g., effortful control, working memory, inhibitory control, cognitive flexibility, proactive control). To correct for multiple comparisons across these models, all resulting *p*‐values were adjusted using the False Discovery Rate procedure. To further analyze the interaction effect we found, simple slope analyses were conducted for each component of cognitive control at each risk type. For the three‐way interaction, Johnson‐Neyman analysis was conducted to identify the age ranges where the effects differed significantly across risk types.

#### Association between BART performance and PRT and NRT, and cognitive control

To examine the relationship between learning performance in the BART experiment and PRT and NRT in real life, we used linear mixed effect models separately for each condition (low and high risk). The number of pumps in each trial served as the dependent variable, with trial number as the main independent variable. For each condition, trial number was included as a fixed effect, while participant‐specific intercepts and slopes for trial number were treated as random effects. This approach allowed us to estimate learning slopes (i.e., how much participants adjusted their number of pumps over trials) while accounting for individual differences in initial pumping behavior. The fixed effect provided the overall learning trend and the random effects provided individual variations in learning trajectories. We then extracted the individual learning slopes for each condition and used these as predictors in linear mixed‐effects models examining associations with risk‐taking behavior (see Figure S2). Unlike the control variable models above, these models included age only as a main effect (not in interactions) since we had no specific hypotheses about age moderating learning slope effects. To explore the association between cognitive control abilities and learning behavior in the BART, we conducted correlation analyses between cognitive control variables and learning slopes in each condition.

## RESULTS

### Descriptive statistics information

Means and standard deviations for all study variables, including covariates, are presented in Table 1. Correlations among these variables are presented in Table 2. For descriptive analyses, differences in PRT and NRT across gender and race/ethnicity were examined, along with correlations with continuous demographic variables in Table 3.

**TABLE 1:** Descriptive statistics for the main study variables.

Variable	*M*	SD	Min	Max
Willingness for Positive Risk‐Taking	0.61	0.21	0.00	1.00
Past Positive Risk‐Taking	0.66	0.17	0.14	1.00
Negative Risk‐Taking	0.28	0.23	0.00	0.93
Working Memory	12.77	5.03	0.00	26.00
Inhibitory Control	0.05	0.06	−0.17	0.22
Cognitive Flexibility	0.10	0.12	−0.24	0.45
Proactive Control	0.57	0.25	−0.19	1.08
Effortful Control	3.06	0.74	1.53	4.73
BART learning in low risk	0.76	0.94	−2.49	3.63
BART learning in high risk	−0.30	0.27	−0.99	0.65
Reward Sensitivity	40.04	4.74	25.00	52.00
Punishment Sensitivity	22.64	4.26	8.00	28.00
Age	15.72	1.74	12.00	18.00

**TABLE 2:** Correlations among main study variables.

Variable	1	2	3	4	5	6	7	8	9	10	11	12
1. Willingness for Positive Risk‐Taking												
2. Past Positive Risk‐Taking	0.31**											
3. Negative Risk‐Taking	0.07	0.21*										
4. Working Memory	0.01	−0.06	−0.14									
5. Inhibitory Control	−0.04	0.1	0.07	−0.01								
6. Cognitive Flexibility	−0.08	−0.09	−0.01	0.03	−0.04							
7. Proactive Control	−0.01	−0.05	−0.26**	0.03	0.02	0.14						
8. Effortful Control	0.14	−0.08	−0.26**	0.26**	0.04	−0.05	0.09					
9. BART learning in low risk	0.08	−0.01	−0.2*	0.1	−0.05	−0.06	0.12	0.03				
10. BART learning in high risk	−0.15	−0.16	0.02	−0.12	−0.23*	0.09	−0.13	0.08	−0.14			
11. Reward Sensitivity	0.29**	0.17	0.13	0.07	−0.02	−0.15	−0.12	0.06	0.13	−0.12		
12. Punishment Sensitivity	−0.06	−0.03	−0.2*	−0.13	0.14	0.01	0.14	−0.24*	−0.02	−0.1	−0.03	
13. Age	0.34***	0.29**	0.41***	0.04	0.21*	0.03	−0.11	−0.21*	−0.16	−0.11	0.18	0.1

*Note*: Pearson correlations are reported.

* *p* < .05.

** *p* < .01.

*** *p* < .001.

**TABLE 3:** Associations between positive and negative risk‐taking with demographic information.

Demographic variable	Willingness PRT	Past PRT	NRT
Age	0.339***	0.286**	0.408***
Puberty Score	0.338***	0.311**	0.264**
SES	0.001	−0.033	−0.102
Gender	*t* = 1.126	*t* = 2.717**	*t* = −1.529
Ethnicity	*F* = 1.947	*F* = 1.588	*F* = 2.524

*Note*: For continuous variables (age, Puberty Score, SES), Pearson correlations are reported. For gender, *t*‐test statistics comparing females vs. males are shown. For Ethnicity, *F*‐statistics from one‐way ANOVA comparing White, Black/Black British, and Asian/Asian British groups are presented.

Abbreviations: NRT, negative risk‐taking; PRT, positive risk‐taking.

** *p* < .01.

*** *p* < .001.

Correlational analyses revealed significant positive associations between age and all risk‐taking measures: willingness for PRT, *r* = .34, *p* < .001, past PRT, *r* = .29, *p* < .01, and NRT, *r* = .41, *p* < .001. Similarly, puberty score was positively correlated with willingness for PRT, *r* = .34, *p* < .001, past PRT, *r* = .31, *p* < .01, and NRT, *r* = .26, *p* < .01. Socioeconomic status (SES) was not significantly associated with any risk‐taking behaviors, all *r*s < .11, *ns*. Independent samples *t*‐tests for gender revealed that females (*M* = 0.70, *SD* = 0.16) demonstrated greater past PRT than males (*M* = 0.61, *SD* = 0.17), *t*(98) = 2.72, *p* < .01. There were no significant differences in willingness for PRT between females (*M* = 0.64, *SD* = 0.22) and males (*M* = 0.59, *SD* = 0.19), *t*(98) = 1.13, *ns*, or NRT between females (*M* = 0.26, *SD* = 0.22) and males (*M* = 0.34, *SD* = 0.25), *t*(98) = −1.53, *ns*. Results from ANOVAs indicated no significant differences across ethnic groups in willingness for PRT, *F*(2, 97) = 1.95, *ns*. Descriptive statistics by ethnicity were as follows: White (*M* = 0.58, *SD* = 0.22), Black/Black British (*M* = 0.63, *SD* = 0.15), and Asian/Asian British (*M* = 0.68, *SD* = 0.19). There were also no significant ethnic differences in past PRT, *F*(2, 97) = 1.59, *ns*, with means of White (*M* = 0.64, *SD* = 0.19), Black/Black British (*M* = 0.72, *SD* = 0.11), and Asian/Asian British (*M* = 0.69, *SD* = 0.13), or NRT, *F*(2, 97) = 2.52, *ns*, with means of White (*M* = 0.28, *SD* = 0.23), Black/Black British (*M* = 0.39, *SD* = 0.31), and Asian/Asian British (*M* = 0.21, *SD* = 0.13).

### Better cognitive control is related with less NRT but no relation with PRT

**FIGURE 1:**
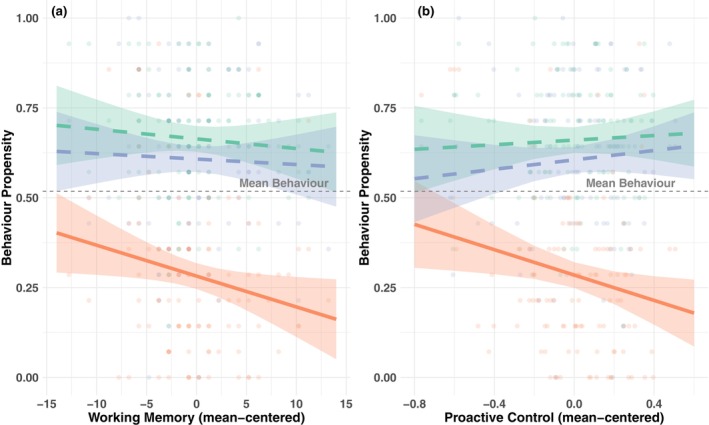
Association between cognitive control task and past positive risk taking and willingness for positive risk taking, and negative risk taking. NRT, negative risk‐taking; PRT, positive risk taking. Solid lines indicate significant associations, and dashed lines indicate nonsignificant associations.

The model for working memory capacity showed significant main effects of risk type, *F*(2, 208) = 132.80, *p* < .001, *p*adj < .001, age, *F*(1, 102) = 37.34, *p* < .001, *p*adj < .001, BAS score, *F*(1, 102) = 6.14, *p* = .015, *p*adj = .037, and BIS score, *F*(1, 102) = 7.37, *p* = .008, *p*adj = .029. Importantly, working memory showed a significant main effect, *β* = −0.009, *SE* = 0.004, *p* = .025, *p*adj = 0.050, indicating a negative association with NRT behavior (Figure 1a). However, no significant interactions were found between working memory and risk type, *F*(2, 208) = 1.03, *p* = .359, *p*adj = .449. The interaction between WM and willingness for PRT was not significant, *β* = 0.007, *SE* = 0.005, *p* = .182, *p*adj = .246, nor was the interaction between WM and past PRT, *β* = 0.006, *SE* = 0.005, *p* = .265, *p*adj = .341. These results suggest that the effect of working memory did not significantly differ across risk types. The three‐way interaction between working memory, risk type, and age was not significant either, *F*(2, 208) = 0.31, *p* = .733, *p*adj = .825.

The model for proactive control showed significant main effects of risk type, *F*(2, 208) = 132.99, *p* < .001, *p*adj < .001, age, *F*(1, 102) = 36.52, *p* < .001, *p*adj < .001, and BIS score, *F*(1, 102) = 6.13, *p* = .015, *p*adj = .037. BAS score effect was not significant after FDR correction, *F*(1, 102) = 4.91, *p* = .029, *p*adj = .063. Importantly, proactive control showed a significant negative main effect, *β* = −0.176, *SE* = 0.073, *p* = .017, *p*adj = .036, indicating that better proactive control was associated with less NRT behavior. Although the overall interaction between proactive control and risk type approached significance, *F*(2, 208) = 3.43, *p* = .034, *p*adj = .067, examination of specific contrasts revealed a significant interaction between proactive control and willingness for PRT, *β* = 0.242, *SE* = 0.100, *p* = .017, *p*adj = .036, while the interaction with past PRT was not significant after correction, *β* = 0.208, *SE* = 0.100, *p* = .038, *p*adj = .064 (Figure 1b). The three‐way interaction between proactive control, risk type, and age was not significant, *F*(2, 208) = 2.99, *p* = .053, *p*adj = .099. To further interpret these interactions, we conducted simple slope analyses for proactive control at each risk type. The results showed a significant negative association between proactive control and NRT, *β* = −0.18, *SE* = 0.07, *t* = −2.40, *p* = .02. Associations with willingness for PRT were numerically positive but not significant, *β* = 0.07, *SE* = 0.07, *t* = 0.89, *p* = .37. Therefore, this interaction effect is driven by a strong negative relation between proactive control and NRT, with no relation to willingness for PRT.

For inhibitory control and cognitive flexibility measures, mixed effect models examining congruency effects of reaction time in the flanker task and switching costs from the cue‐switching paradigm revealed no significant associations with risk‐taking behaviors. ANOVA results are in Table S2 and model specifications are in Table S3. However, supplementary correlation analyses revealed additional patterns for flanker task performance measures. Overall flanker accuracy showed a significant negative correlation with NRT, *r* = −.24, *p* = .012, indicating that better interference control is associated with NRT. Additionally, the flanker congruency effect for accuracy demonstrated a marginally significant positive correlation with NRT, *r* = .19, *p* = .051, suggesting that individuals with greater susceptibility to interference may be more prone to NRT. Results are presented in Figure S1. Overall, better cognitive control task performance demonstrated consistent associations with reduced NRT but showed no distinctive relationships with PRT.

### Effortful control is related with less NRT and more willingness for PRT

From the model with effortful control, the analysis revealed significant main effects of risk type, *F*(2, 208) = 126.33, *p* < .001, *p*adj < .001, and age, *F*(1, 102) = 33.49, *p* < .001, *p*adj < .001. Both types of PRT were reported significantly more frequently than NRT, with past PRT, *β* = 0.367, *SE* = 0.025, *p* < .001, *p*adj < .001, showing a slightly stronger effect than willingness for PRT, *β* = 0.310, *SE* = 0.025, *p* < .001, *p*adj < .001. Age was positively associated with risk‐taking behavior, β = 0.043, *SE* = 0.011, *p* < .001, *p*adj < .001, indicating that older participants reported more risk‐taking behavior propensity overall. BAS score showed a significant positive relationship with risk‐taking behavior, *β* = 0.006, *SE* = 0.002, *F*(1, 102) = 5.68, *p* = .019, *p*adj = .039, indicating that higher reward sensitivity was related to increased risk‐taking. BIS score showed a significant negative relationship with risk‐taking behavior, *β* = −0.007, *SE* = 0.003, *F*(1, 102) = 6.63, *p* = .011, *p*adj = .032, indicating that higher punishment sensitivity was related to decreased risk‐taking.

**FIGURE 2:**
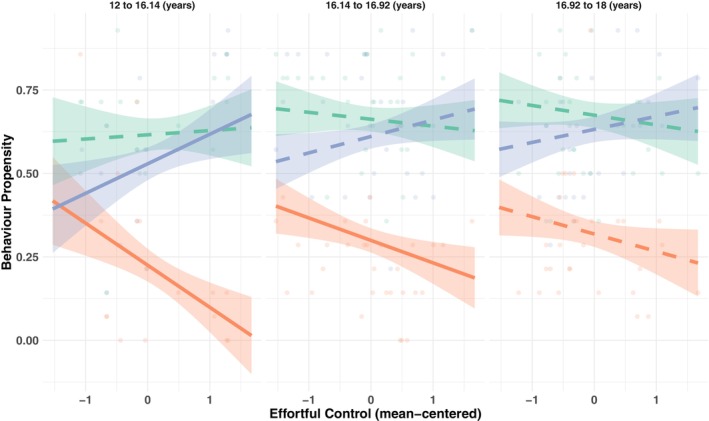
Association between effortful control and past positive risk‐taking and willingness for positive risk‐taking, and negative risk‐taking across age ranges. NRT, negative risk‐taking; PRT, positive risk‐taking. Solid lines indicate significant associations, and dashed lines indicate non‐significant associations.

Significant interactions were found between effortful control and risk type, *F*(2, 208) = 7.45, *p* < .001, *p*adj = .003, *η* ^2^p = .07. The effect of effortful control differed significantly between NRT and willingness for PRT, *β* = 0.130, *SE* = 0.034, *p* < .001, *p*adj < .001, and was not significant between NRT and past PRT, *β* = 0.059, *SE* = 0.034, *p* = .080, *p*adj = .119. A significant three‐way interaction between effortful control, risk type, and age was also found, *F*(2, 208) = 4.30, *p* = .015, *p*adj = .037, *η* ^2^p = .04, suggesting that the relationships between effortful control and the different types of risk‐taking changed with age. The interaction effect was significant for both NRT versus past PRT, *β* = −0.053, *SE* = 0.022, *t*(208) = −2.456, *p* = .015, *p*adj = .034, and NRT versus willingness for PRT, *β* = −0.056, *SE* = 0.022, *t*(208) = −2.619, *p* = .009, *p*adj = .028. These negative coefficients indicate that as age increased, the difference in the effect of effortful control on PRT (both past behavior and willingness) compared to NRT decreased. Specifically, for younger adolescents, there was a greater divergence in how effortful control related to willingness for PRT versus NRT. As age increased, this divergence became less pronounced (Figure 2).

To further examine this three‐way interaction, Johnson‐Neyman analysis was conducted to identify the age ranges where effortful control effects differed significantly across risk types. For NRT, effortful control had significant effects (*p* < .05) for participants younger than 16.92 years. Within the observed age range of 12–18 years, this indicates significant negative associations between effortful control and NRT for participants aged 12.00–16.92 years, with effects becoming nonsignificant for participants aged 16.92–18.00 years. Higher effortful control was associated with lower engagement in NRT among younger adolescents. For willingness for PRT, effortful control effects were significant (*p* < .05) for participants younger than 16.14 years. Within the observed age range, effortful control was positively associated with willingness for PRT for participants aged 12.00–16.14 years. Effects became nonsignificant for participants older than 16.14 years. Thus, higher effortful control was associated with greater willingness for PRT among younger adolescents. For past PRT, no significant interval was identified, indicating that effortful control effects did not reach statistical significance at any age level.

Overall, effortful control was associated with adaptive risk‐taking patterns in younger adolescents (approximately ages 12–16 years), specifically with lower negative risks and greater willingness to engage in positive risks. The effects diminish as participants approach late adolescence (approximately ages 16–18 years).

### Learning in low‐risk condition is related to less NRT but no relation with PRT

#### Overall BART performance

**FIGURE 3:**
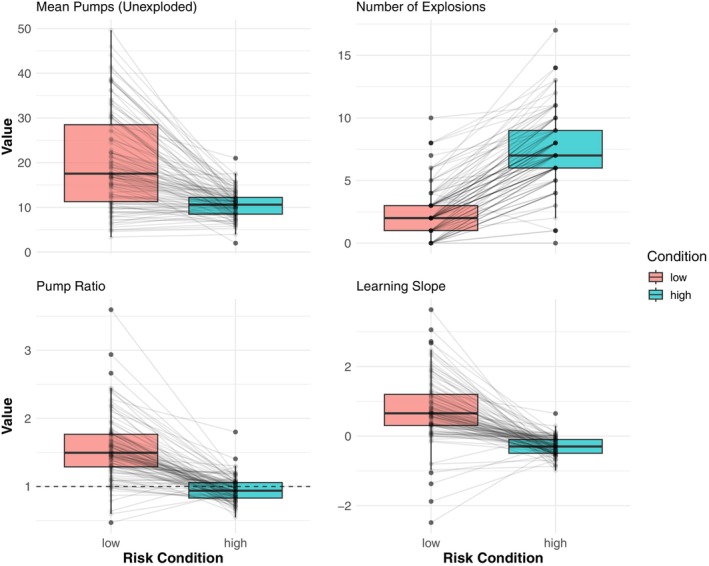
BART performance measures across low‐ and high‐risk conditions. Each panel displays boxplots with individual data points and connecting lines showing within‐participant changes between conditions.

To examine if participants learned explosion probabilities of the two balloons, we compared participants' behavior across low‐ and high‐risk conditions in a two BART using four measures: *mean* number of pumps for unexploded balloons, the number of explosions, pump ratio (i.e., the number of pumps in the first half of the task divided by one in the second half of the task), and learning slope (Figure 3). Paired‐samples *t*‐tests were conducted for each measure.

First, participants pumped significantly more in the low‐risk condition (*M* = 20.11, *SD* = 10.74) than the high‐risk condition (*M* = 10.61, *SD* = 3.06), *t* = 9.92, *p* < .001, suggesting they were willing to inflate balloons to a greater extent when the perceived risk was lower. Also, participants experienced significantly more explosions in the high‐risk condition (*M* = 7.57, *SD* = 2.88) compared to the low‐risk condition (*M* = 2.92, *SD* = 1.88), *t* = −21.64, *p* < .001, despite more conservative pumping behavior in the former condition. The pump ratio, defined as the number of inflations in the second half of the task divided by the number of inflations in the first half, was significantly higher for low‐risk (*M* = 1.54, *SD* = 0.48) than high‐risk balloons (*M* = 0.94, *SD* = 0.18), *t* = 12.88, *p* < .001. The *mean* ratio above 1 for the low‐risk balloon suggests that participants learned to maximize their opportunity to earn points in that condition. Conversely, for high‐risk balloons, the *mean* ratio below 1 indicated decreasing inflations and demonstrated adaptive learning to avoid risk in that condition. This clear separation from the reference line at 1.0 in both conditions further supports this adaptive learning pattern, *t*(107) = 12.08, *p* < .001, *t*(107) = −2.98, *p* = .004, respectively. Lastly, learning slope, which shows the change in the number of pumps across trials for each participant, demonstrated significantly different patterns between conditions. Low‐risk trials showed a positive trend across trials (*M* = 0.76, *SD* = 0.94), whereas high‐risk trials showed a negative trend across trials (*M* = −0.30, *SD* = 0.27), *t(107)* = 10.80, *p* < .001. This indicates that participants learned to increase their pumping behavior in low‐risk conditions while simultaneously learning to decrease their pumping behavior in high‐risk conditions. Overall, these findings suggest that participants successfully learned to distinguish between the two risk levels and adapted their strategies accordingly.

To investigate the association between learning behavior in the BART and participants' risk‐taking propensity in daily life, we obtained learning slopes for each participant for the low‐ and high‐risk conditions. To do this, we conducted a linear mixed model analysis on the number of pumps separately for the low‐ and high‐risk conditions. As we expected opposite linear trends in pumping behavior in each risk condition, we explored the association between low‐ and high‐risk learning slopes on behavioral propensity separately.

Only in the low‐risk condition, significant interactions were found between learning slope and risk type, *F*(2, 212) = 3.87, *p* = .022, *p*adj = .033, *η* ^2^p = .035. Specifically, the interaction between learning slope and willingness for PRT was significant, *β* = 0.145, *SE* = 0.055, *p* = .009, *p*adj = .021, as was the interaction between learning slope and past PRT, *β* = 0.116, *SE* = 0.055, *p* = .036, *p*adj = .046. These interactions indicate that the effect of learning slope differed between NRT and both past and willingness for PRT.

**FIGURE 4:**
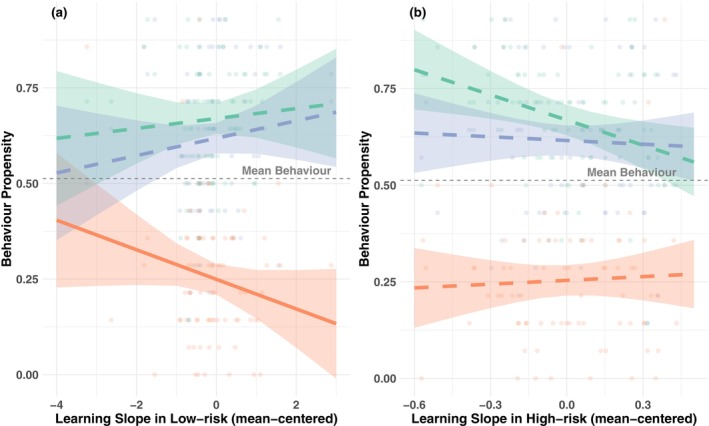
Association between learning slope in the BART and past positive risk‐taking and willingness for positive risk‐taking, and negative risk‐taking in a low‐risk and high‐risk condition. NRT, negative risk‐taking; PRT, positive risk‐taking. Solid lines indicate significant associations, and dashed lines indicate nonsignificant associations.

To further interpret these interactions, we conducted simple slope analyses for learning slope at each risk type. The results showed a significant negative association between learning slope and NRT, *β* = −0.084, *SE* = 0.041, *t* = −2.058, *p* = .040. Associations with both willingness and past PRT were numerically positive but not significant, *β* = 0.061, *SE* = 0.041, *t* = 1.499, *p* = .135, and *β* = 0.032, *SE* = 0.041, *t* = 0.793, *p* = .428, respectively. Therefore, faster learning in the low‐risk condition of the BART was associated with less NRT in real life (Figure 4).

Last, to investigate the association between learning behavior in the BART and participants' cognitive control abilities, we examined correlations between learning slopes for each participant in the low‐ and high‐risk conditions and four cognitive control measures: working memory, inhibitory control, cognitive flexibility, and proactive control. Given the multiple comparisons involved, we applied False Discovery Rate correction.

Inhibitory control, as measured by congruency effects of reaction time in the flanker task (where smaller values indicate better performance), showed a significant association with learning slope in the high‐risk condition, *r* = −.23, *p* = .015, *padj* = .118. While this association was significant before correction, it did not survive FDR correction. All other correlations between cognitive control measures and learning slopes in both conditions were not significant (*r* = −.13 to .12).

## DISCUSSION

The current study aimed to expand understanding of the underlying cognitive mechanisms that drive adolescent adaptive risk‐taking by addressing four key research questions. First, how do different components of cognitive control (working memory, cognitive flexibility, inhibitory control, and proactive control) relate to adolescent adaptive risk‐taking? Second, does effortful control, as a measure of cognitive control applied in emotionally charged, real‐life contexts, show distinct associations with positive and negative risk‐taking? Third, does learning ability in risk‐taking experiments relate to real‐world adaptive risk‐taking? Finally, do cognitive control abilities associate with better learning in risk‐taking?

First, our hypothesis that better cognitive control capacity would relate to adaptive risk‐taking that is less NRT and more PRT, received only partial support, showing that while cognitive control measures (working memory, proactive control) are associated with reduced NRT, they showed no distinctive associations with PRT. Specifically, working memory showed a negative association with NRT, aligning with previous research (Peeters et al., 2017; Romer et al., 2011). Especially poorer working memory in adolescents is related to impulsivity, resulting in decreased ability to suppress momentary urges and this leads to adolescent NRT (Khurana et al., 2012; Romer et al., 2011). However, working memory was not associated with PRT in our study, which is contrary to our expectations and surprising given that the complex behaviors often associated with PRT require holding information in mind. Further research is needed to clarify how working memory might relate to risk‐taking. In line with this, proactive control demonstrated a significant negative association with NRT. Proactive control may contribute to adaptive emotional regulation, a key factor in risk‐taking, as youth at risk for anxiety showed fewer anxiety issues when they had higher levels of proactive control, suggesting a protective role in emotional regulation (Valadez et al., 2022). Furthermore, as proactive control relates to planning and future‐oriented thinking (Andrews‐Hanna et al., 2011; Mahy & Munakata, 2015), through anticipation and preparation for future cognitive demands, proactive control may help adolescents anticipate aversive outcomes in NRT cases.

Surprisingly, we did not find a clear association between inhibitory control and NRT which has been robustly observed in previous studies, although our supplementary analysis on the flanker accuracy showed a strong negative relationship with NRT. This discrepancy may be due to task characteristics, as we used the congruency effect of reaction time from the Flanker task, which focuses on interference control rather than tasks that tap impulse suppression or involve withholding a prepotent response under emotional arousal, which have often been used in previous research (Shulman et al., 2016). Similarly, cognitive flexibility showed no association with risk‐taking, although we expected it would be related since adolescents need to adapt their behavior in risk‐taking situations where outcomes are uncertain. As with inhibitory control, this may be due to the limitations of the cued‐task switching task we used, which did not adequately capture flexibility under emotionally demanding situations. Recent research has shown that affective flexibility paradigms using emotion‐evoking stimuli (i.e. faces with emotional valence) were significantly correlated to resilience, while neutral stimuli (i.e. even and odd digits) were not (Rademacher et al., 2023). Together, these unexpected findings suggest the need to further examine how these cognitive control components might relate to adolescent risk‐taking, particularly through experimental measures within emotional arousal contexts.

Second, effortful control exhibited the most pronounced diverging pattern between PRT and NRT. Not only was greater effortful control associated with reduced NRT as in prior studies (King et al., 2013; Peeters et al., 2017; Wang et al., 2015), but it was also associated with greater PRT. Although this pattern had never been documented in the context of PRT, it is consistent with previously reported associations between greater effortful control and both increased prosocial behaviors and reduced aggression or disruptive behaviors in children (Zhou et al., 2004). Effortful control may show the strongest associations with NRT and PRT because it captures cognitive control in emotionally and motivationally charged situations, and thus links with emotion regulation. In other words, effortful control may best capture cognitive control engagement to counter the influence of the reward system. Indeed, effortful control can buffer the effects of negative contexts such as poverty, neighborhood conditions, and parental mental health, which can lead adolescents to engage in NRT (Lengua et al., 2008). Greater effortful control may reduce the impact of negative emotional traits on externalizing behaviors, such as aggression, in adolescents (Oldehinkel et al., 2007), and contribute to better stress management (Oldehinkel et al., 2011), which may be important in PRT scenarios, as they often involve stress and anxiety. Adolescents with strong effortful control may be more capable of taking positive risks because they can manage the initial discomfort or anxiety that comes with stepping outside their comfort zone (e.g., reaching out to people in a new class), and resist the immediate appeal of easier and potentially more immediately rewarding options (e.g., stick with familiar friends from a previous class).

Interestingly, the divergent pattern of associations of effortful control with NRT and PRT was most conspicuous in younger adolescents and diminished in older adolescents. In parallel, adolescents engaged in more NRT behavior with age, regardless of their cognitive control. This may be due to the increasing impact of external factors such as environmental context, peer pressure, and social influences, as adolescents gain more independence from the family unit (Willoughby et al., 2014). The mere availability or accessibility of risky situations plays a crucial role in determining whether an individual engages in risky behavior (Fryt et al., 2021; Gerrard et al., 2008). One meta‐analysis found that adolescents generally do not take more risks than children on behavioral decision‐making tasks, contrary to predictions of neurodevelopmental models, but adolescents show more real‐life risk‐taking than children and the author proposed that opportunity may be a key factor explaining why adolescents engage in more real‐life risk‐taking than children (Defoe et al., 2015). Thus, greater opportunities for risk‐taking in older adolescents may leave less room for the influence of cognitive control (Shulman et al., 2016). Similarly, although availability of opportunities plays an important role in all types of risk, this role may be especially acute for PRT, which may also require important resources, such as access to extracurricular activities or advanced educational programmes that may not be equally available to all youth. Thus, access to these resources and opportunities likely affects the extent to which willingness to engage in PRT can be translated into concrete actions, regardless of one's effortful control. This may explain why associations with effortful control were observed for willingness to engage in PRT rather than past PRT engagement, and why the magnitude of these associations was not as high as for NRT.

Given that effortful control emerged as the strongest effect on PRT, we conducted a post‐hoc exploratory analysis to examine whether this relation was consistent across effortful control subcomponents or driven by specific subcomponents (i.e., attention, activation, and inhibitory control). Supplementary analysis revealed that activation control showed no significant relation with PRT. Results are shown in Figure S1. This finding is surprising given that activation control (i.e., the ability to perform actions despite a tendency to avoid them) is highly relevant to PRT situations where the impulse is to avoid but action is required. We further explored this effect and found that older adolescents reported poorer activation control (*r* = −.22, *p* = .020), while there was no age effect in other subcomponents. The activation control items primarily assess procrastination behaviors (e.g., “I put off working on projects until right before they're due”). Older adolescents may have reported poor activation control due to increased academic demands and pressures. Also, it should be noted that in our study, the effortful control questionnaire consisted of a short set of survey items within the temperament instrument, which was not sufficient to distinguish subcomponents empirically. Future research should utilize the full questionnaire to investigate each subcomponent more thoroughly, and given that age appears to play a role, larger samples in each age group would be beneficial for understanding these developmental patterns in effortful control.

At first glance, these findings may speak against the dual‐process model assumption that the cognitive control and reward systems necessarily operate in opposition, as both systems may favor PRT. Note that we interpreted the results regarding effortful control through the lens of the dual‐process model, given that effortful control overlaps significantly with cognitive control in terms of its conceptual framework and neural correlates (Zhou et al., 2012). Although the two systems may support the overall risky goal it could be the case that these systems may still operate in opposition at the level of specific behaviors, but with opposing influence on PRT compared to NRT. Specifically, PRT often involves engaging in effortful and sometimes daunting behaviors that do not result in immediate reward (e.g., signing up for a challenging course or volunteering for a stressful presentation). In such cases, effortful control may facilitate goal‐directed behavior toward long‐term benefits, while the reward system may pull adolescents away from these demanding activities in favor of more immediately gratifying alternatives. Conversely, for NRT, effortful control typically inhibits impulsive behaviors while the reward system drives engagement in immediately rewarding but potentially harmful activities. Thus, effortful control may favor PRT and reduce NRT, while the reward system may favor NRT and reduce PRT, creating a complex interplay where the same neurocognitive systems have opposing effects on different types of risk‐taking at different levels.

In addition, although the dual‐process models regard heightened reward sensitivity only as a motivation for NRT (e.g., trying out harmful substances), it may also serve as an important driving force for adolescent development (e.g., trying new sports; Crone & Dahl, 2012; Romer et al., 2017; Duell & Steinberg, 2021). Similarly, increased sensitivity to social rewards may prompt reckless behaviors for peer acceptance, but it can also inspire prosocial behaviors and foster new friendships. Thus, although our findings do not directly speak to the dynamics of the reward and control systems in PRT, these dynamics are likely to be especially complex and will deserve direct investigation in the future. For example, testing the interaction between risk type and reward sensitivity would provide valuable insights into how the dual system model explains different types of risk‐taking behavior.

The third hypothesis that learning capability on the BART would be associated with less NRT and more PRT received partial support. Learning slope in low‐risk conditions, as measured in BART, showed a diverging pattern of association with NRT and PRT, though only the negative association with NRT reached statistical significance. While our findings cannot establish causality, greater learning of action outcomes in risky situations may help adolescents avoid harmful behaviors while better recognizing attainable beneficial outcomes. This pattern manifests differently in NRT versus PRT scenarios. In NRT situations, adolescents might experiment impulsively but cease after experiencing negative consequences. In PRT contexts, they may learn that uncertain but rewarding experiences promote growth, encouraging future beneficial risk‐taking. These findings align with the Life Span Wisdom model, which emphasizes the role of experience and learning during adolescence (Romer et al., 2017). This model suggests that exploratory risk‐taking under ambiguity peaks in adolescence, while impulsive risk‐taking and risk‐taking under known probabilities decline from childhood to adulthood.

Notably, learning slope showed significant positive correlations with total points in low‐risk condition. This suggests that in low‐risk conditions, some degree of risk‐taking (i.e., more pumping across trials despite the risk) is necessary for optimal performance. However, we found no significant correlations between total points and learning slope in the high‐risk condition. This difference may arise because in high‐risk situations, sharply decreasing pump numbers is not always adaptive, as it could prevent obtaining potential rewards. While we attempted to reduce stochasticity to simplify learning patterns, a simple linear trend (decreasing in high‐risk and increasing in low‐risk conditions) may not adequately capture the learning process in this task. Future research could employ more sophisticated learning parameters to better capture these learning dynamics and their relation to real‐life risk‐taking among adolescents.

Last, our fourth hypothesis that higher cognitive control abilities would be associated with better learning performance on the BART was not supported, a finding inconsistent with previous studies (Blair et al., 2018; Ogilvie et al., 2020). This discrepancy may stem from our task design, which used distinct low‐ and high‐risk blocks, potentially reducing working memory demands compared to mixed‐block designs (e.g., Blair et al., 2018). Nevertheless, non‐significant trends suggested that better working memory (*r* = .10 in low risk; *r* = −.12 in high risk) and proactive control (*r* = .12 in low risk; *r* = −.13 in high risk) may be linked to more adaptive learning (i.e., increased pumping in the low‐risk condition and decreased pumping in the high‐risk condition). Results are shown in Table S5. Notably, these are the same cognitive control functions that were related to reduced NRT in our study. Future research with a sufficient sample size may benefit from using mediation analysis to explore the mechanisms through which cognitive control influences real‐world risk‐taking.

However, these findings must be interpreted with caution. A critical limitation is that our adapted BART task did not relate to PRT, suggesting it fails to capture the complexity of real‐world PRT. This reflects broader concerns about the ecological validity of laboratory tasks in predicting real‐world behavior (De Groot, 2020). The BART's focus on monetary outcomes differs fundamentally from PRT, which involves pursuing uncertain social, academic, or personal growth opportunities like trying out for a team or taking a challenging course (Duell & Steinberg, 2019). Thus, future research must develop experimental paradigms that better capture the essence of PRT in adolescent development.

Despite being the first study, to our knowledge, to compare experimental measures of cognitive control to both PRT and NRT behaviors, several additional limitations should be acknowledged. While we decided not to measure willingness for NRT due to ethical considerations and its impulsive nature, future research might explore indirect methods such as examining peer influence (e.g., “Would you participate if your friends were involved?”) or using carefully constructed hypothetical scenarios that do not directly encourage negative behaviors. Additionally, the self‐report measure of risk‐taking presents inherent limitations due to lifestyle confounders. This is particularly evident in PRT, which encompasses various activities (social, academic, physical) that vary among individuals based on personal preferences, accessibility, and social context. While NRT behaviors are relatively homogeneous across individuals, PRT demonstrates considerable variance in its characteristics and manifestations.

Future investigations could benefit from a multi‐method approach that complements traditional questionnaires with experimental paradigms specifically designed to capture PRT. Using these methods in conjunction would help minimize lifestyle confounders and mitigate social desirability biases, leading to a more robust understanding of PRT. Additionally, future studies with larger samples could conduct more comprehensive psychometric validation (e.g., Patterson et al., 2022) to further establish the robustness of the PRT scale across different populations. This would also enable investigation of how demographic factors such as gender, SES and cultural contexts shape adolescent risk‐taking patterns. This is especially important, as PRT is heavily influenced by family environment, available resources, and support systems (Fryt et al., 2021). Research incorporating gender, diverse SES backgrounds and cultural settings could provide valuable insights into how demographic and environmental factors interact with cognitive processes in shaping both PRT and NRT behaviors.

In conclusion, this study provides novel insights into cognitive mechanisms associated with adolescent adaptive risk‐taking, revealing distinct patterns of association for PRT and NRT. While cognitive control (working memory, proactive control) and learning on the BART were primarily associated with reduced NRT, effortful control showed a unique pattern of association, linking to both lower negative and greater PRT. Risk‐taking, whether positive or negative, plays an important role in adolescent development, helping them step out of their comfort zone, embrace challenges, and foster independence. Indeed, not all NRT is entirely harmful but a certain level of risk‐taking is beneficial as adolescents transition toward independent adulthood (Duell & Steinberg, 2019). The key is guiding adolescents to avoid severely harmful risks (e.g., riding with an intoxicated driver) while encouraging positive ones. The present findings suggest that effortful control may play a key role in achieving adaptive balance. To develop beneficial interventions for adolescents, approaches should not only focus on reducing NRT but also actively promote PRT. The current research contributes to this goal by expanding our understanding of how different cognitive control components relate to risk‐taking.

## AUTHOR CONTRIBUTIONS

Hyeji Lee led multiple aspects of the project: conceptualization, data curation, methodology, formal analysis, visualization, and project administration. Lee also took primary responsibility for writing the original draft and participated in the review process. Stella Haffner was responsible for data curation and contributed to the manuscript's review and editing. Bonnie Auyeung contributed to the project's conceptualization and participated in the review and editing of the manuscript. Nicolas Chevalier supervised the project and developed its methodology. He contributed to the conceptualization and co‐authored the original draft, also participating in the review and editing. All authors reviewed and approved the final manuscript.

## FUNDING INFORMATION

This research was supported by a grant from the School of Philosophy, Psychology and Language Sciences at the University of Edinburgh. Hyeji Lee was supported by a scholarship from the National Institute for International Education. Nicolas Chevalier was supported by Leverhulme Trust grant RPG‐2021‐235. Bonnie Auyeung was supported by the European Union's Horizon 2020 research and innovation programme under the Marie Skłodowska‐Curie grant agreement no. 813546, the Baily Thomas Charitable Fund TRUST/VC/AC/SG/469207686 and the UK Economic and Social Research Council (ES/W001519/1) during the course of this work.

## CONFLICT OF INTEREST STATEMENT

The authors have no relevant financial or non‐financial interests to disclose.

## ETHICS STATEMENT

This study was conducted under the approval of the University of Edinburgh ethics board (reference number: 421‐2122/6), granted on October 11, 2022.

## PATIENT CONSENT

All participants provided informed consent to participate in this study. For participants under 16 years of age, parental consent was also obtained.

## Supporting information


**Figure S1.** Detailed correlation matrix across control variables and risk‐taking.
**Table S1.** Model comparison (all variables vs. reduced).
**Table S2.** ANOVA table results.
**Table S3.** Mixed model results.
**Table S4.** A linear mixed model for extracting learning rate (learning under uncertainty).
**Figure S2.** Individual learning trajectories with fixed effect line.
**Figure S3.** Correlation matrix across BART measures and risk‐taking.
**Table S5.** Correlations between cognitive control and BART performance.
**Figure S4.** Overview of the online study.
**Figure S5.** Cued task‐switching paradigm.

## Data Availability

The data necessary to reproduce the analyses presented here are publicly accessible, as are the supplementary materials necessary to attempt to replicate the findings. The primary analytic code necessary to reproduce the analyses presented in this work is publicly available. Any additional custom code and raw data are available from the corresponding author upon reasonable request. Analyses were not pre‐registered. Data, code, and materials of this research are available at the following URL: https://osf.io/nf2qx/.
