# Supplementary material for: Beyond risk reduction: Exploring the relation of cognitive control with adolescent positive and negative risk‐taking
Source: J Res Adolesc. 2025 Nov 24;35(4):e70103. doi: 10.1111/jora.70103 (PMC12644316; doi:10.1111/jora.70103)
Supplement: Supplementary file 1 — Figure S1. Detailed correlation matrix across control variables and risk‐taking. Table S1. Model comparison (all variables vs. reduced). Table S2. ANOVA table results. Table S3. Mixed model results. Table S4. A linear mixed model for extracting learning rate (learning under uncertainty). Figure S2. Individual learning trajectories with fixed effect line. Figure S3. Correlation matrix across BART measures and risk‐taking. Table S5. Correlations between cognitive control and BART performance. Figure S4. Overview of the online study. Figure S5. Cued task‐switching paradigm. [file JORA-35-0-s001.docx]

**Beyond Risk Reduction: Exploring the Relation of Cognitive Control with Adolescent Positive and Negative Risk Taking**

***Supplementary Materials***

**Figure S1: Detailed Correlation Matrix across Control Variables and Risk Taking­­­­­­**


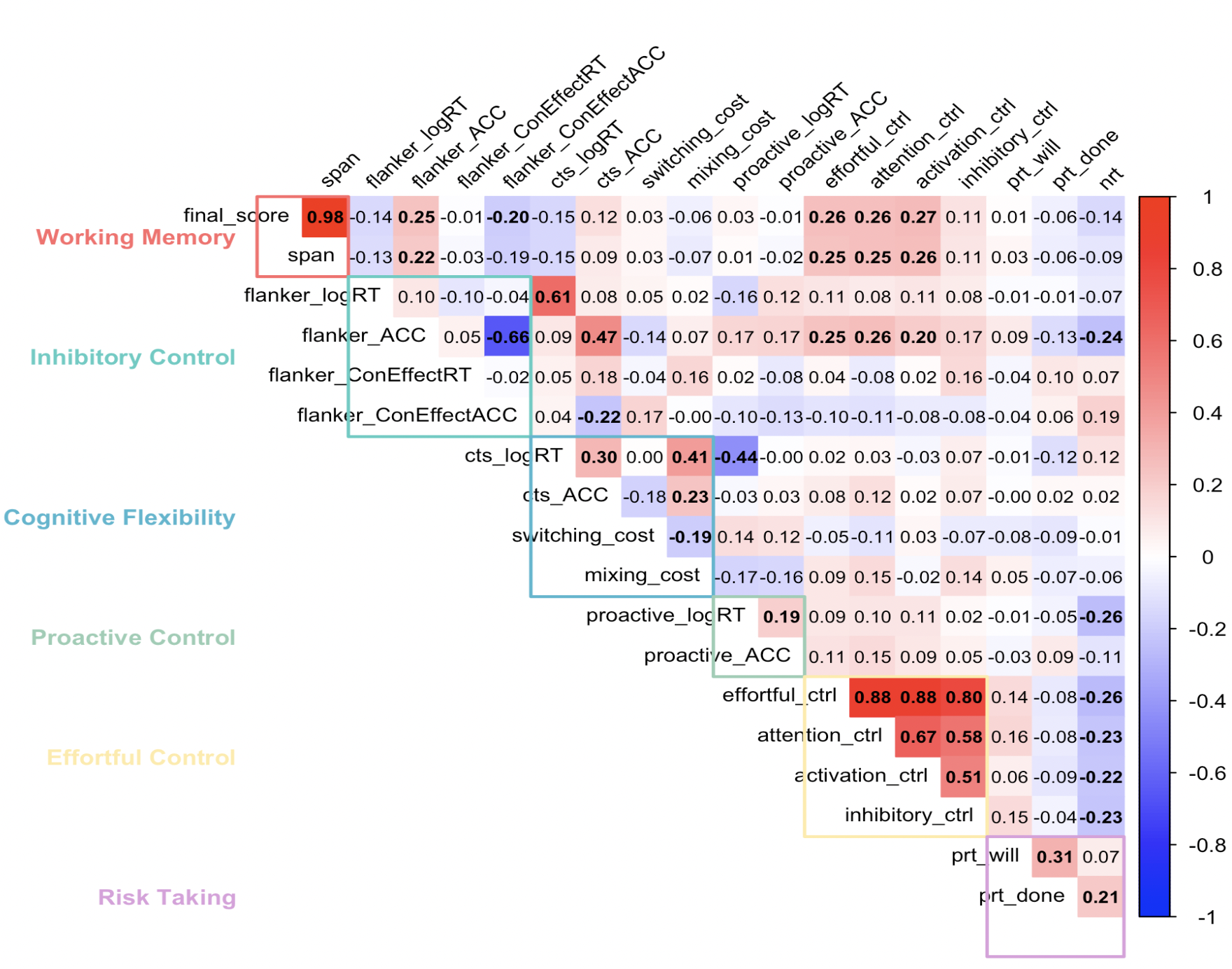


Note. RT = response time; ACC = accuracy; ctrl = control; CTS = Cued Task Switching; final_score = number of correct trials in Backward Digit Span task; span = maximum span achieved in Backward Digit Span task; effortful_ctrl = average score of all subcomponents (attention_ctrl, activation_ctrl, and inhibitory_ctrl); prt_will = willingness for positive risk taking; prt_done = past positive risk taking behaviour; nrt = past negative risk taking behaviour. Bold values indicate p < .05.

***Within-Domain Correlations.*** Examination of within-domain correlations revealed strong internal consistency across measures. Within the effortful control domain, the effortful control composite, attention control, activation control, and inhibitory control were highly intercorrelated (rs = .513-.876, all ps < .001), indicating strong internal consistency. Within inhibitory control as measured by the flanker task, overall accuracy and congruency effect accuracy were strongly negatively correlated (r = -.656, p < .001), suggesting that better overall performance was associated with smaller interference effects.

Within cognitive flexibility, reaction time and accuracy were positively correlated (r = .299, p = .002), indicating that participants who responded more slowly in the task-switching paradigm also had lower accuracy, suggesting individual differences in overall cognitive processing efficiency rather than a speed-accuracy trade-off. Switching cost and mixing cost showed a significant negative relationship (r = -.193, p = .045), suggesting that individuals who experienced greater costs when switching between tasks (higher switching costs) tended to have lower costs associated with performing in mixed-task contexts compared to single-task blocks (lower mixing costs), potentially reflecting different cognitive strategies or compensatory mechanisms. Within proactive control, reaction time and accuracy were significantly correlated (r = .194, p = .044), indicating that participants who responded more slowly when comparing proactive versus reactive cue conditions also demonstrated lower accuracy, reflecting individual differences in the efficiency of proactive cognitive control.

Among risk taking measures, positive risk taking willingness and past positive risk taking variety scores correlated significantly (r = .310, p = .001), indicating consistency between willingness to engage in positive risk behaviour and actual past engagement. Past positive risk taking showed a significant association with negative risk taking frequency (r = .214, p = .026), suggesting some overlap between positive and negative risk taking tendencies.

***Cross-Domain Correlations***

Cross-domain correlations indicated significant associations between different control constructs. Working memory (backward digit span performance) showed significant correlations with the effortful control composite (r = .257, p = .007), attention control (r = .256, p = .007), and activation control (r = .274, p = .004), but not with inhibitory control (r = .112, p = .248). Working memory also correlated with flanker accuracy (rs = .219-.248, ps < .05). Additionally, flanker accuracy correlated positively with cognitive flexibility accuracy (r = .474, p < .001), suggesting shared variance across inhibitory control and cognitive flexibility domains.

Effortful control measures showed consistent negative correlations with negative risk taking frequency. The effortful control composite (r = -.263, p = .006), attention control (r = -.229, p = .017), activation control (r = -.217, p = .024), and inhibitory control (r = -.227, p = .018) all demonstrated significant inverse relationships with negative risk taking behaviours. Inhibitory control as measured by the flanker task also related to risk taking behaviours, with overall flanker accuracy correlating negatively with negative risk taking (r = -.241, p = .012). The flanker congruency effect for accuracy showed a marginally significant positive correlation with negative risk taking (r = .188, p = .051), indicating that individuals with greater susceptibility to interference may be more prone to negative risk taking. Proactive control demonstrated a significant negative association with negative risk taking (r = -.258, p = .007).

Among risk taking measures, positive risk taking willingness and past positive risk taking variety scores correlated significantly (r = .310, p = .001), indicating consistency between willingness to engage in positive risk behaviours and actual past engagement. Past positive risk taking showed a significant association with negative risk taking frequency (r = .214, p = .026), suggesting some overlap between positive and negative risk taking tendencies.

**Table S1: Model Comparison (All variables vs. Reduced)**

| Predictor | AIC Reduced | BIC Reduced | χ² | df | p |
| --- | --- | --- | --- | --- | --- |
| Effortful Control | -158.400 | -97.903 | 3.789 | 4.000 | 0.435 |
| Proactive Control | -149.190 | -88.701 | 3.446 | 4.000 | 0.486 |
| Working Memory | -142.690 | -82.200 | 3.722 | 4.000 | 0.445 |
| Inhibitory Control | -139.570 | -79.082 | 2.993 | 4.000 | 0.559 |
| Cognitive Flexibility | -138.910 | -78.419 | 3.008 | 4.000 | 0.556 |

*Note*. The reduced model, specified as **behaviour propensity ~ cognitive_measure * risk_type * age + BAS_total + BIS_sum + (1 | Participant)**, was directly compared to a full model that additionally incorporated socioeconomic status, gender, and race/ethnicity (with race/ethnicity dummy-coded using White as the reference group). An initial analysis with the full model revealed that these demographic variables did not significantly account for variability in the dependent variable. In accordance with statistical criteria and the principle of parsimony and given that these variables are peripheral to our core hypotheses, the reduced model was selected for further analysis. As a result, the final model focuses on reward sensitivity and punishment sensitivity as covariates, in line with previous research demonstrating their relevance to both negative and positive risk taking among adolescents (Duell & Steinberg, 2020)

**Table S2: ANOVA table results**

| **Effect** | **Effortful Control** | **Working Memory** | **Inhibitory Control** | **Proactive control** | **Cognitive Flexibility** |
| --- | --- | --- | --- | --- | --- |
| Control variables | F(1,102)=0.6, p=0.440, p_adj=0.521 | F(1,102)=3.45, p=0.066, p_adj=0.119 | F(1,102)=0.00, p=0.992,  p_adj=0.992 | F(1,102)=0.34, p=0.563, p_adj=0.650 | F(1,102)=2.18, p=0.143, p_adj=0.217 |
| Risk Type | F(2,208)=126.33***, p=<.001, p_adj=<.001*** | F(2,208)=132.8***, p=<.001, p_adj=<.001*** | F(2,208)=128.28***, p=<.001, p_adj=<.001*** | F(2,208)=132.99***, p=<.001, p_adj=<.001*** | F(2,208)=130.78***, p=<.001, p_adj=<.001*** |
| Age | F(1,102)=33.49***, p=<.001, p_adj=<.001*** | F(1,102)=37.34***, p=<.001, p_adj=<.001*** | F(1,102)=37.66***, p=<.001, p_adj=<.001*** | F(1,102)=36.52***, p=<.001, p_adj=<.001*** | F(1,102)=39.47***, p=<.001, p_adj=<.001*** |
| BAS | F(1,102)=5.68*, p=0.019, p_adj=0.045* | F(1,102)=6.14*, p=0.015, p_adj=0.037* | F(1,102)=5.25*, p=0.024, p_adj=0.051 | F(1,102)=4.91*, p=0.029, p_adj=0.063 | F(1,102)=4.75*, p=0.032, p_adj=0.065 |
| BIS | F(1,102)=6.63*, p=0.011, p_adj=0.037* | F(1,102)=7.37**, p=0.008, p_adj=0.029* | F(1,102)=6.03*, p=0.016, p_adj=0.038* | F(1,102)=6.13*, p=0.015, p_adj=0.037* | F(1,102)=6.56*, p=0.012, p_adj=0.037* |
| Control variables  × Risk Type | F(2,208)=7.45***, p=<.001, p_adj=0.003** | F(2,208)=1.03, p=0.359, p_adj=0.449 | F(2,208)=0.54, p=0.585, p_adj=0.716 | F(2,208)=3.43*, p=0.034, p_adj=0.067 | F(2,208)=0.07, p=0.937, p_adj=0.958 |
| Control variables  × Age | F(1,102)=0.07, p=0.794, p_adj=0.871 | F(1,102)=0, p=0.980, p_adj=0.980 | F(1,102)=1.30, p=0.257, p_adj=0.379 | F(1,102)=0.75, p=0.388, p_adj=0.472 | F(1,102)=0.03, p=0.853, p_adj=0.914 |
| Risk Type × Age | F(2,208)=1.1, p=0.335, p_adj=0.430 | F(2,208)=1.78, p=0.171, p_adj=0.240 | F(2,208)=1.72, p=0.182, p_adj=0.276 | F(2,208)=1.19, p=0.305, p_adj=0.404 | F(2,208)=1.43, p=0.241, p_adj=0.328 |
| Control variables  × Risk Type × Age | F(2,208)=4.3*, p=0.015, p_adj=0.037* | F(2,208)=0.31, p=0.733, p_adj=0.825 | F(2,208)=0.44, p=0.647, p_adj=0.759 | F(2,208)=2.99, p=0.053, p_adj=0.099 | F(2,208)=0.11, p=0.897, p_adj=0.939 |

Note. p_adj = p-values adjusted for multiple comparisons using the False Discovery Rate (FDR) correction. *p < .05, **p < .01, ***p < .001.

**Table S3: Mixed model results**

| **Effect** | **Effortful Control** | **Working Memory** | **Inhibitory Control** | **Proactive control** | **Cognitive Flexibility** |
| --- | --- | --- | --- | --- | --- |
| (Intercept) | B=0.291***, t=15.932, p=<.001, p_adj=<.001*** | B=0.282***, t=15.491, p=<.001, p_adj=<.001*** | B=0.28***, t=15.098, p=<.001, p_adj=<.001*** | B=0.285***, t=15.723, p=<.001, p_adj=<.001*** | B=0.282***, t=15.38, p=<.001, p_adj=<.001*** |
| Control variables | B=-0.075**, t=-3.005, p=0.003, p_adj=0.009** | B=-0.009*, t=-2.249, p=0.025, p_adj=0.050* | B=0.06, t=0.178, p=0.859, p_adj=0.872 | B=-0.176*, t=-2.401, p=0.017, p_adj=0.037* | B=-0.091, t=-0.599, p=0.550, p_adj=0.688 |
| Risk Type(prt_done) | B=0.367***, t=14.761, p=<.001, p_adj=<.001*** | B=0.382***, t=15.092, p=<.001, p_adj=<.001*** | B=0.383***, t=14.888, p=<.001, p_adj=<.001*** | B=0.376***, t=15.1, p=<.001, p_adj=<.001*** | B=0.382***, t=14.981, p=<.001, p_adj=<.001*** |
| Risk_Type(prt_will) | B=0.31***, t=12.488, p=<.001, p_adj=<.001*** | B=0.326***, t=12.872, p=<.001, p_adj=<.001*** | B=0.323***, t=12.56, p=<.001, p_adj=<.001*** | B=0.321***, t=12.887, p=<.001, p_adj=<.001*** | B=0.325***, t=12.768, p=<.001, p_adj=<.001*** |
| Age | B=0.043***, t=4.008, p=<.001, p_adj=<.001*** | B=0.054***, t=4.943, p=<.001, p_adj=<.001*** | B=0.053***, t=4.789, p=<.001, p_adj=<.001*** | B=0.049***, t=4.666, p=<.001, p_adj=<.001*** | B=0.053***, t=4.931, p=<.001, p_adj=<.001*** |
| BAS | B=0.006*, t=2.384, p=0.019, p_adj=0.040* | B=0.006*, t=2.479, p=0.015, p_adj=0.036* | B=0.005*, t=2.292, p=0.024, p_adj=0.049* | B=0.005*, t=2.216, p=0.029, p_adj=0.055 | B=0.005*, t=2.179, p=0.032, p_adj=0.058 |
| BIS | B=-0.007*, t=-2.575, p=0.011, p_adj=0.032* | B=-0.007**, t=-2.715, p=0.008, p_adj=0.024* | B=-0.006*, t=-2.455, p=0.016, p_adj=0.037* | B=-0.007*, t=-2.475, p=0.015, p_adj=0.036* | B=-0.007*, t=-2.561, p=0.012, p_adj=0.032* |
| Control variables × Risk Type(prt_done) | B=0.059, t=1.76, p=0.080, p_adj=0.127 | B=0.006, t=1.117, p=0.265, p_adj=0.375 | B=0.143, t=0.306, p=0.760, p_adj=0.814 | B=0.208*, t=2.084, p=0.038, p_adj=0.067 | B=-0.069, t=-0.327, p=0.744, p_adj=0.814 |
| Control variables × Risk Type(prt_will) | B=0.13***, t=3.855, p=<.001, p_adj=<.001*** | B=0.007, t=1.338, p=0.182, p_adj=0.266 | B=-0.33, t=-0.705, p=0.481, p_adj=0.624 | B=0.242*, t=2.417, p=0.017, p_adj=0.037* | B=-0.063, t=-0.298, p=0.766, p_adj=0.814 |
| Control variables × Age | B=0.034*, t=2.12, p=0.035, p_adj=0.062 | B=0.001, t=0.458, p=0.647, p_adj=0.755 | B=0.097, t=0.598, p=0.550, p_adj=0.688 | B=0.049, t=1.353, p=0.177, p_adj=0.263 | B=0.016, t=0.212, p=0.832, p_adj=0.867 |
| Risk Type(prt_done) × Age | B=-0.016, t=-1.111, p=0.268, p_adj=0.375 | B=-0.028, t=-1.885, p=0.061, p_adj=0.101 | B=-0.027, t=-1.792, p=0.075, p_adj=0.121 | B=-0.022, t=-1.52, p=0.130, p_adj=0.198 | B=-0.025, t=-1.689, p=0.093, p_adj=0.144 |
| Risk Type(prt_will) × Age | B=0.004, t=0.296, p=0.767, p_adj=0.814 | B=-0.013, t=-0.844, p=0.400, p_adj=0.548 | B=-0.007, t=-0.489, p=0.626, p_adj=0.742 | B=-0.007, t=-0.522, p=0.602, p_adj=0.727 | B=-0.011, t=-0.736, p=0.463, p_adj=0.611 |
| Control variables × Risk Type(prt_done) × Age | B=-0.053*, t=-2.456, p=0.015, p_adj=0.036* | B=-0.003, t=-0.76, p=0.448, p_adj=0.603 | B=-0.082, t=-0.369, p=0.712, p_adj=0.814 | B=-0.109*, t=-2.218, p=0.028, p_adj=0.054 | B=-0.034, t=-0.333, p=0.740, p_adj=0.814 |
| Control variables × Risk Type(prt_will) × Age | B=-0.056**, t=-2.619, p=0.009, p_adj=0.028* | B=-0.001, t=-0.2, p=0.842, p_adj=0.867 | B=0.125, t=0.559, p=0.577, p_adj=0.708 | B=-0.098*, t=-1.998, p=0.047, p_adj=0.080 | B=0.012, t=0.116, p=0.908, p_adj=0.908 |

Note. p_adj = p-values adjusted for multiple comparisons using the False Discovery Rate (FDR) correction. *p < .05, **p < .01, ***p < .001, prt_will = willingness for positive risk taking; prt_done = past positive risk taking; negative risk taking is reference level of Risk Type variable

**Complementary Analysis on the BART**

**Table S4: A linear mixed model for extracting learning rate (Learning under uncertainty)**

| **Low-risk condition** | | | | |
| --- | --- | --- | --- | --- |
| **Terms** | **b** | ***SE* b** | ***t*** | ***p*** |
| *(Intercept)* | *15.36* | *1.48* | *10.37* | *< .001 **** |
| Trial | 0.76 | 0.09 | 8.20 | < .001 *** |
| **Random Effects** |  |  |  |  |
| (Residual) | 157.48 |  |  |  |
| (Intercept) | 234.21 |  |  |  |
| Trial | 0.91 |  |  |  |
| ICC | 0.598 |  |  |  |
| N_Subjects_ | 108 |  |  |  |
| **High-risk condition** | | | | |
| **Terms** | **b** | ***SE* b** | ***t*** | ***p*** |
| *(Intercept)* | 15.33 | 0.45 | 33.88 | *< .001 **** |
| Trial | -0.30 | 0.03 | -11.08 | < .001 *** |
| **Random Effects** |  |  |  |  |
| (Residual) | 21.58 |  |  |  |
| (Intercept) | 21.69 |  |  |  |
| Trial | 0.08 |  |  |  |
| ICC | 0.50 |  |  |  |
| N_Subjects_ | 108 |  |  |  |

*Note*. Each model includes 108 participants and 43,327 observations. The model equation in lmer syntax is as follows: Pump ~ Trial + (Trial | Participant) in each risk condition. The participant-specific random effects for “Trial” were extracted and these were combined with the fixed effect of “Trial” to calculate each participant's total learning slope. The results were transformed into a data frame, with participant IDs and learning slopes clearly labelled. This process provided individual learning slopes, reflecting how participants adapted their behaviour across trials.

**­**

**Figure S2: Individual Learning Trajectories with Fixed Effect Line**


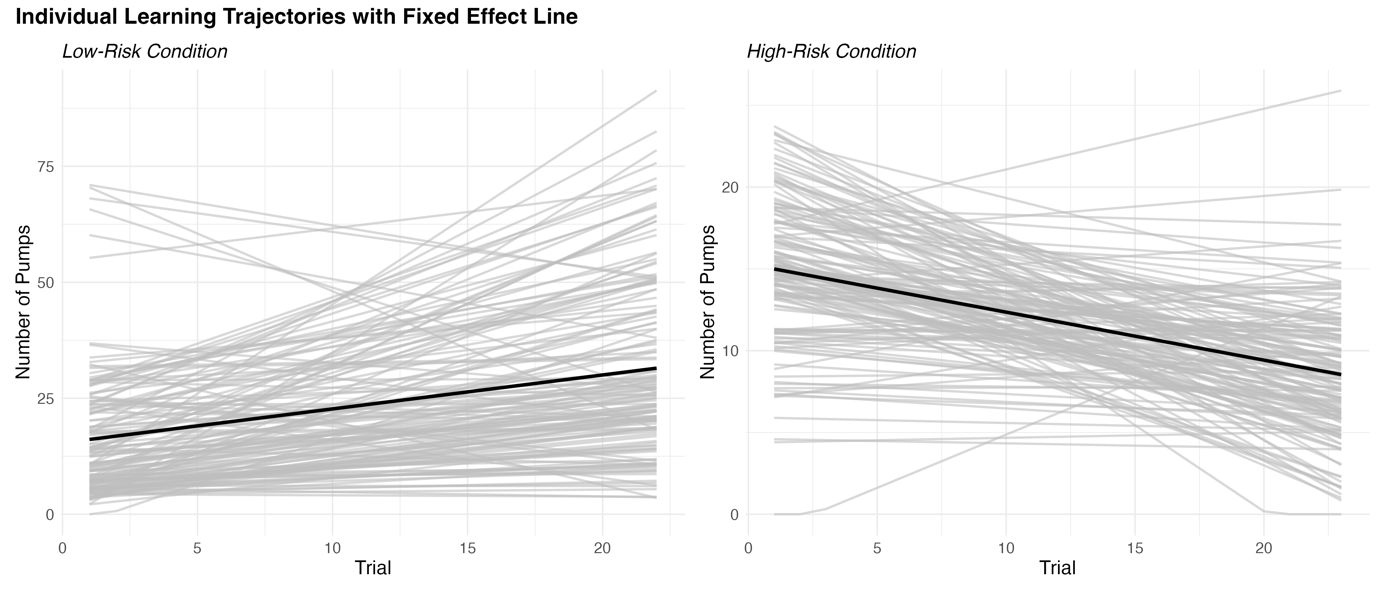


**Figure S3: Correlation matrix across BART measures and Risk Taking**

**
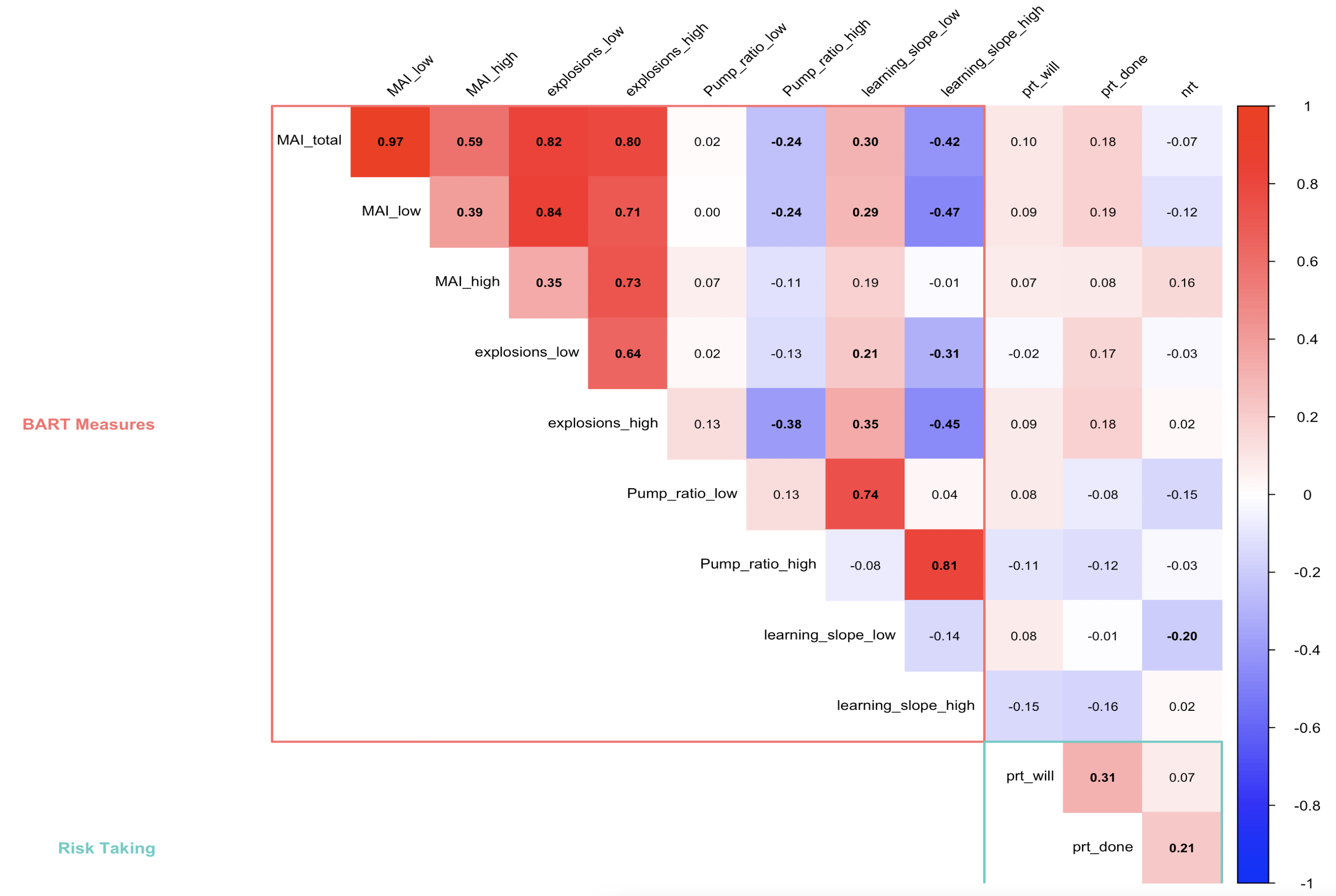
**

*Note.* Mean Adjusted Inflation (MAI) is an average number of pumps on unexploded balloons, representing total points earned as well as risk taking propensity. Explosions is the number of exploded balloons. Pump ratio is the number of inflations in second half divided by first half, indicating learning adaption. Learning slope is individual rate of pump adjustments over tirals, derived from linear mixed-effects models.

| Variable | Working Memory | Flanker RT | Flanker ACC | Flanker Con Effect RT | Flanker Con Effect ACC | CTS RT | CTS ACC | Switching Cost | Proactive RT | Proactive ACC |
| --- | --- | --- | --- | --- | --- | --- | --- | --- | --- | --- |
| Mean Pumps (high) | 0.040 | -0.224 | -0.126 | 0.055 | 0.032 | -0.206 | -0.094 | 0.054 | -0.152 | -0.075 |
| Mean Pumps (low) | 0.154 | -0.130 | -0.135 | -0.047 | 0.112 | -0.341** | -0.093 | -0.047 | 0.047 | -0.025 |
| Explosions (high) | 0.168 | -0.253* | -0.225 | 0.082 | 0.087 | -0.390*** | -0.214 | 0.067 | -0.048 | -0.147 |
| Explosions (low) | 0.087 | -0.164 | -0.259* | -0.067 | 0.219 | -0.363*** | -0.164 | -0.125 | 0.085 | -0.089 |
| Pump Ratio (high) | -0.079 | 0.054 | 0.044 | -0.284* | 0.196 | 0.051 | -0.140 | -0.075 | -0.033 | -0.017 |
| Pump Ratio (low) | 0.068 | -0.050 | 0.033 | -0.132 | -0.069 | -0.056 | -0.237 | 0.001 | 0.087 | -0.057 |
| Learning Slope (high) | -0.124 | 0.087 | 0.011 | -0.234 | 0.131 | 0.150 | -0.075 | -0.097 | -0.129 | 0.010 |
| Learning Slope (low) | 0.100 | -0.051 | -0.066 | -0.054 | -0.029 | -0.163 | -0.221 | -0.047 | 0.117 | -0.036 |
| Note. * p < .05. ** p < .01. *** p < .001 (FDR corrected). | | | | | | | | | | |

**Table S5: Correlations between cognitive control and BART performance**

**Figure S4: Overview of the online study**


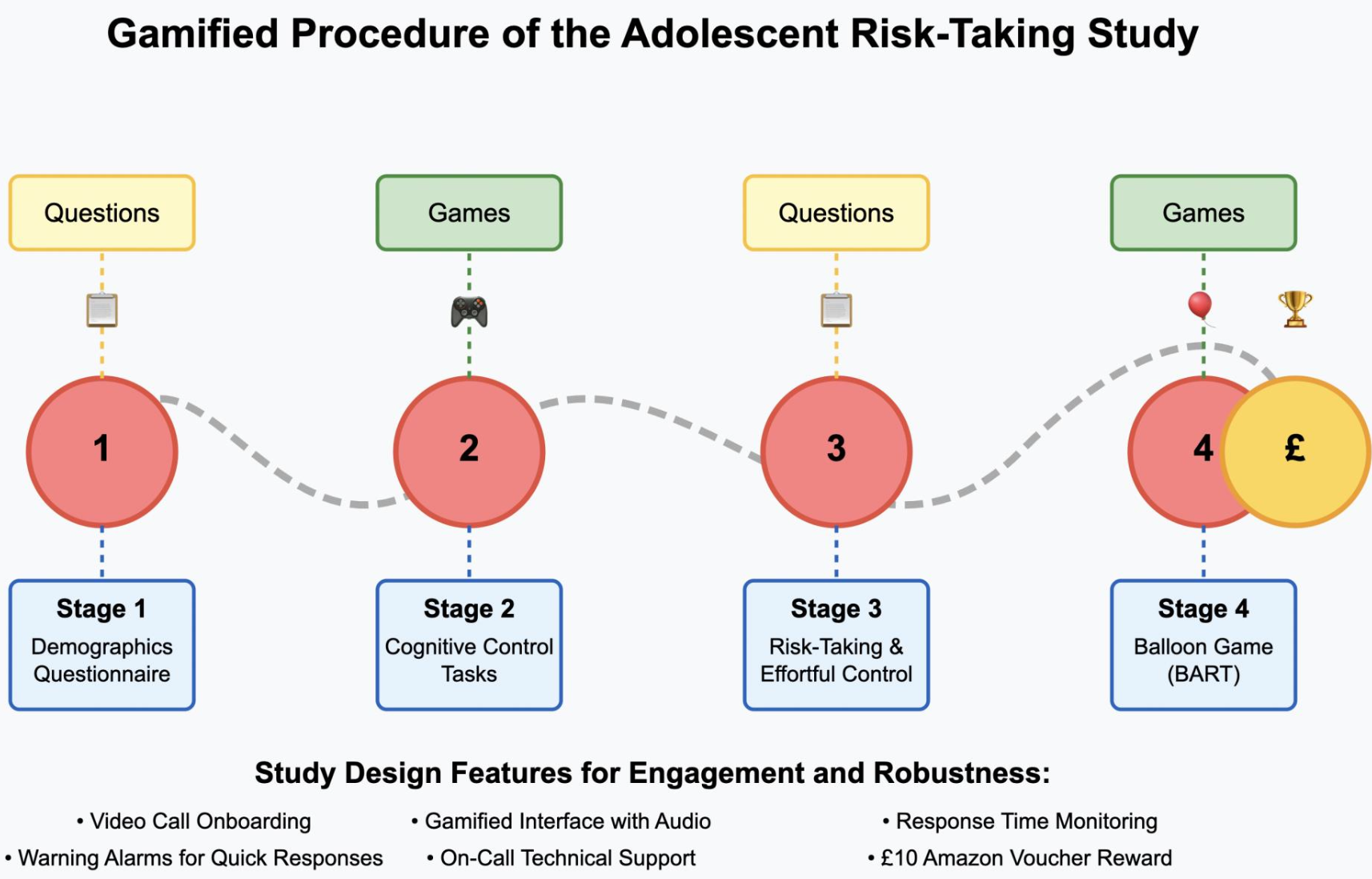


**Figure S5: Cued task-switching paradigm**


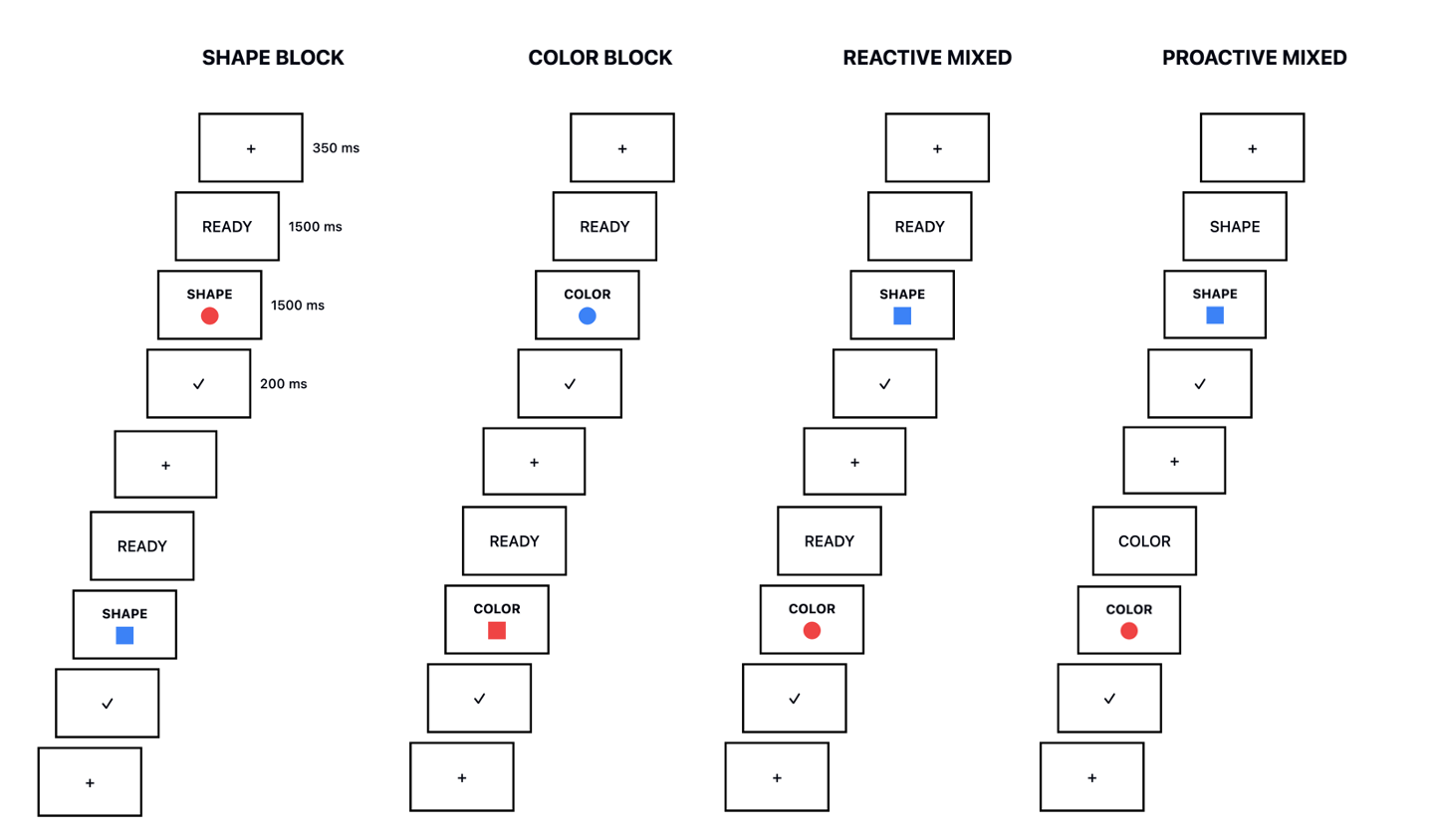


*Note.* Trial structure: 350ms fixation → 1500ms ready/cue → 1500ms stimulus → 200ms feedback. Single blocks: 16 trials each (4 practice + 12 test), Mixed blocks: 48 trials each.
